# Caffeic Acid Regulates High Glucose-Induced Inflammatory Responses in BV-2 Microglial Cells: Implications for Purinergic Signaling

**DOI:** 10.1007/s11064-026-04893-6

**Published:** 2026-10-31

**Authors:** Milagros Fanny Vera Castro, Charles Elias Assmann, Bianca Vedoin Copês Rambo, Mairin Schott, Marcylene Vieira da Silveira, Vitor Bastianello Mostardeiro, Adriel Antonio Schirmann, Pâmela de Almeida Milioni, Michele Goulart dos Santos, Vera Maria Melchiors Morsch, Roselia Maria Spanevello, Maria Rosa Chitolina Schetinger

**Affiliations:** Toxicological Biochemistry, Center of Natural and Exact Sciences, Federal University of Santa Maria, University Campus, Camobi District, Santa Maria, RS 97105-900 Brazil

**Keywords:** Phenolic compounds, Microglia, Neuroinflammation, Cellular hyperglycemia, Purinergic signaling, Diabetes mellitus

## Abstract

High glucose-induced microglial activation contributes to inflammatory processes associated with diabetic complications. Thus, the effects of caffeic acid (CA) on inflammatory responses and purinergic signaling parameters in high glucose-challenged BV-2 microglial cells were investigated. A) Oxidative stress; b) Enzymatic activities of Ecto-nucleoside triphosphate diphosphohydrolase (E-NTPDase), Ecto-5-nucleotidase (E-5’-NT), and Adenosine deaminase (ADA); c) Density of P2X7R, A1R, and A2AR purinoceptors; d) Inflammatory markers (IBA-1, NLRP3, and IL-1β); and e) Apoptosis and cell cycle were assessed. Treatment with CA (50 and 75 µM, 24 h) in cells exposed to high glucose medium (GL 25 mM, 48 h) increased viability and reduced nitric oxide (NO), reactive species (RS), and thiobarbituric acid reactive substances (TBARS) levels compared to high glucose-challenged cells. CA also restored ATP and ADP hydrolysis and reduced ADA activity. Furthermore, CA increased the density of the A1R while reversing the high glucose-induced increase in P2X7R and A2AR densities. Moreover, CA treatment attenuated the increase in IL-1β density in high glucose medium, whereas only CA (75 µM) treatment reduced NLRP3 and IBA-1 densities. Besides, this phenolic compound decreased apoptosis and promoted cell cycle progression by increasing the proportion of cells in the S and G2/M phases. Overall, findings suggest that CA attenuates high glucose-induced inflammatory and oxidative alterations in BV-2 microglial cells, besides modulating purinergic enzyme activities and purinoceptor density. These results support the potential involvement of purinergic signaling in the cellular effects of CA and provide possible mechanistic insights into its antioxidant and anti-inflammatory actions under high glucose conditions.

**Supplementary Information:**

The online version contains supplementary material available at https://doi.org/10.1007/s11064-026-04893-6.

## Highlights

Caffeic acid (CA) reduced oxidative stress parameters in BV-2 cells exposed to high glucose.

CA decreased the density of purinoceptors associated with inflammation and increased the levels of receptors associated with neuroprotection.

CA decreased inflammatory markers and cell death in high glucose-challenged microglial cells.

CA could be a phenolic compound further investigated to attenuate high glucose-induced effects found in the diabetic state.

**Supplementary Information:**

The online version contains supplementary material available at https://doi.org/10.1007/s11064-026-04893-6.

## Introduction

Diabetes mellitus (DM) is a chronic metabolic disease characterized by hyperglycemia, which leads to severe damage and complications over time [1–3]. Thus, one of the major complications of DM associated with damage to brain structure and function is diabetic encephalopathy (DE) [4–7]. Considering the brain as one of the main targets of hyperglycemia [1, 8, 9], a growing body of evidence points to high glucose levels as the main factor associated with the development of DE and subsequent cognitive impairment [8, 10, 11]. Clinical manifestations of DE include attention deficit, motor impairment, cognitive and memory dysfunction, behavioral disturbances, severe cerebral thrombosis, and stroke [11–15].

Microglial activation is an essential and beneficial response to noxious stimuli in the central nervous system (CNS); however, under pathological conditions, overactivation of microglial cells can cause brain tissue damage, contributing to cognitive impairment and the pathogenesis of several neurodegenerative diseases [8, 9, 16–20]. Thus, microglial activation, oxidative stress, cytokine release, and apoptosis, responsible for inflammatory responses induced by high glucose [17, 20–22], are considered key features of neurodegenerative processes associated with DM [21, 22]. Consequently, these inflammatory responses promote the progression of DE [6, 20, 23, 24]. In light of these considerations, the restoration of microglial homeostasis, particularly under dysfunctional states, represents a promising therapeutic strategy to prevent or attenuate the progression of neurodegenerative processes [18, 19, 25, 26]. Therefore, controlling microglia-mediated neuroinflammatory responses may contribute to preventing further neuronal injury and slowing the progression of cognitive deficits in DE.

In this context, among the wide range of biological processes in which the purinergic system is involved, its crucial role in mechanisms related to neuroinflammation and neurodegeneration is noteworthy [27–31]. The purinergic signaling pathway involves nucleotides such as ATP, ADP, and AMP, and the nucleoside adenosine (Ado); moreover, this network also includes P1 receptors (activated by Ado) and P2 receptors, subdivided into P2X and P2Y types (activated mainly by ATP and ADP). The biological outcomes are partially determined by the expression of these receptors, as well as the extracellular concentrations of their ligands [32–34]. Furthermore, the concentration of these ligands is regulated by the enzymatic chain initially coordinated by the activity of Ecto-nucleoside triphosphate diphosphohydrolases (E-NTPDases), which hydrolyze ATP and/or ADP to AMP, and Ecto-nucleotide pyrophosphatase/phosphodiesterases (E-NPPs), which can break down ATP directly to AMP. AMP is subsequently hydrolyzed to Ado by the action of Ecto-5’-nucleotidase (E-5’-NT), and finally, Ado is degraded by adenosine deaminase (ADA) to produce inosine [33, 35–38].

Particularly, high extracellular ATP concentrations act as a pro-inflammatory signaling molecule, and can activate P2X7R, one of the main P2X receptor subtypes [33, 39], whose activation has been associated with pro-inflammatory responses, neurodegeneration, and cell death [29, 40, 41]. In this context, accumulating evidence has shown the core pathophysiological role of the ionotropic P2X7R in CNS disorders. Notably, P2X7R is highly expressed in microglia, where its activation has been implicated in microglial reactivity, IL-1β release, and neuroinflammation. Moreover, P2X7R stimulation is recognized as one of the classical pathways involved in NLRP3 inflammasome activation [11, 18, 29, 40, 42].

On the other hand, Ado, the main metabolite of the enzymatic degradation of ATP, deserves special attention due to its importance in the CNS, where it plays a multifactorial role during neuroinflammatory processes [29, 43]. This molecule exerts anti-inflammatory, immunosuppressive, and neuromodulatory effects, thereby contributing to neuroprotection depending on its binding to its receptors [36, 38, 43, 44]. Among the four known Ado receptors, A2AR and A2BR are coupled to Gs proteins and can increase intracellular cAMP levels by the activation of adenylyl cyclase (AC), whereas A1R and A3R are coupled to Gi proteins, which can inactivate this enzyme and lower the levels of cAMP [45]. Ado signaling has been shown to participate in the regulation of glucose metabolism and insulin resistance in DM and obesity [46]. Furthermore, among Ado metabotropic receptors, A1R and A2AR subtypes are known to mediate relevant physiological and pathological actions of Ado in the brain. Generally, A1R has been associated with a more neuroprotective profile, whereas A2AR to a more neuroinflammatory action [33, 38, 47].

In order to identify new adjuvant therapies that can attenuate the neurological complications associated with DM, several treatment strategies utilizing phenolic compounds have been the subject of investigation. Among these molecules, caffeic acid (CA) has been reported to exhibit a broad spectrum of pharmacological activities, including antioxidant [48–50], anti-inflammatory [51], hypoglycemic [50–53], insulin secretagogue [54], anticancer [48, 55–57], and neuroprotective [49, 56, 58, 59]. Moreover, research has shown the therapeutic potential of CA against brain damage, memory impairment, and cognitive decline [59–61]. Notably, our previous study extended these findings by demonstrating that CA attenuated oxidative stress, neuroinflammation, purinergic and cholinergic dysfunction, and cognitive impairment in streptozotocin (STZ)-induced diabetic rats [59], supporting its therapeutic potential against DM-associated neurodegeneration. Collectively, these findings have strengthened the rationale for investigating CA not only as a bioactive compound with intrinsic pharmacological properties but also as a promising adjuvant to conventional therapeutic strategies for DM and its neurological complications.

Building upon our previous findings from the in vivo DM model, wherein CA was demonstrated to alleviate the neurodegenerative process [59], the cellular mechanisms underlying these neuroprotective effects remain incompletely understood. Therefore, the present study was designed to explore the effects of this phenolic acid on oxidative-inflammatory responses and purinergic signaling in BV-2 microglial cells exposed in vitro to a high glucose environment. This approach was designed to provide insights into the cellular mechanisms that may contribute to the neuroprotective effects previously observed in vivo. Importantly, neurodegeneration was not directly assessed in the present study; rather, we focused on microglia-mediated inflammatory responses, which may contribute to neuronal injury and neurodegenerative processes associated with DM.

## Materials and Methods

### Chemicals, Reagents, and Equipment

All chemicals and reagents used in this investigation, including caffeic acid (CA), anhydrous D-glucose, adenosine triphosphate (ATP), adenosine diphosphate (ADP), adenosine monophosphate (AMP), adenosine (Ado), trichloroacetic acid (TCA), 2,7 dichlorodihydrofluorescein diacetate (DCFH-DA), malachite green, ammonia molybdate, and polyvinyl alcohol were purchased from Sigma-Aldrich Inc. (St. Louis, MO, USA). All other reagents used in the experiments, unless otherwise stated, were of analytical grade and of the highest purity.

The materials used in cell culture procedures were acquired from Gibco^®^ (Thermo Fisher Scientific, Grand Island, NY, USA), Vitrocell Embriolife (Campinas, SP, Brazil), Kasvi (São José dos Pinhais, PR, Brazil), and Corning Inc. (Corning, NY, USA). The fluorimetric and spectrophotometric measurement analyses were carried out using a SpectraMax^®^ i3 Multimode Plate Reader (Molecular Devices, Sunnyvale, CA, USA). For flow cytometry, the FITC Annexin V and Propidium Iodide (PI) staining kit was acquired from BD Biosciences (San Diego, CA, USA). Protocols were carried out using a FACSCanto™ II Flow Cytometer (BD Biosciences) apparatus. Data collected from flow cytometry were subsequently analyzed using the FlowJo vX.0.7 software (Tree Star, Inc., Ashland, OR, USA).

### Microglial Cell Culture Procedures and Treatments

**Fig. 1:**
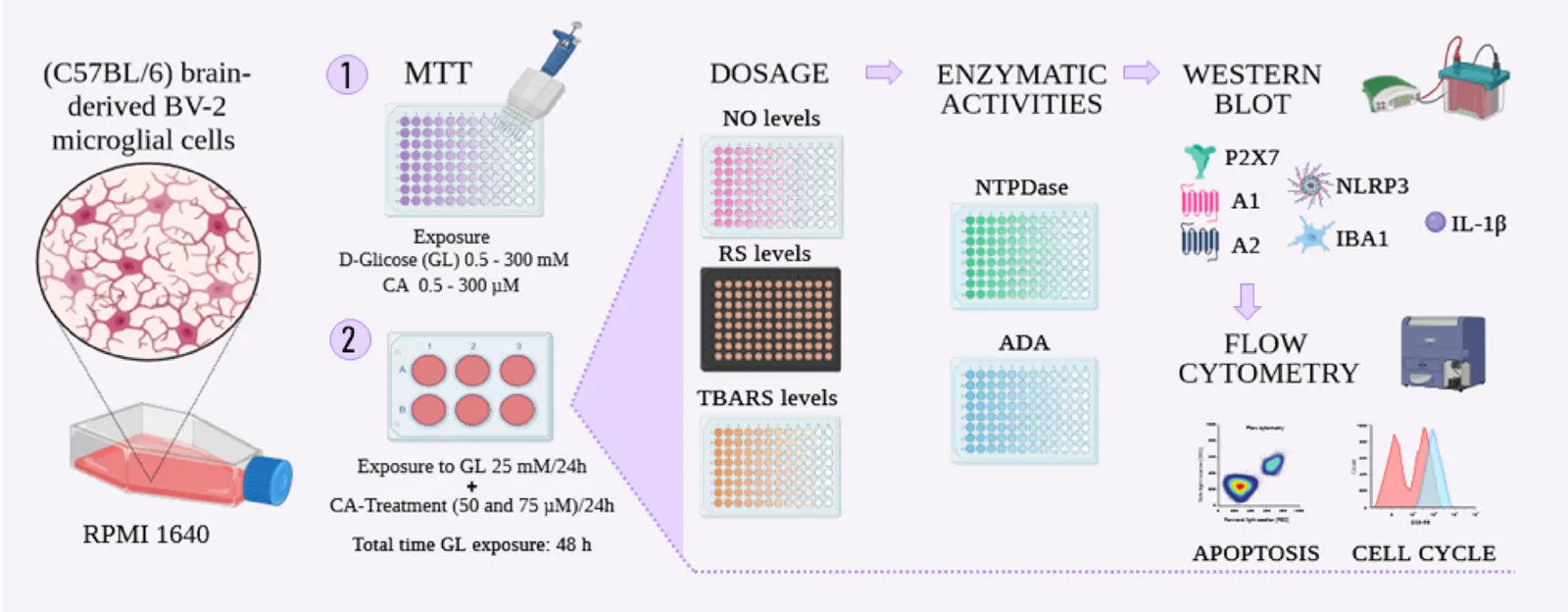
Experimental protocol of the treatment with selected CA (50 and 75 µM) concentrations for 24 h in a model in vitro of BV-2 microglial cells exposed to high glucose (25 mM) medium during 48 h. CA, Caffeic Acid; GL, High glucose; NO, Nitric oxide; RS, Reactive species; TBARS, Thiobarbituric acid reactive species; NTPDase, Ecto-nucleoside triphosphate diphosphohydrolase; ADA, Adenosine deaminase. This image was created with templates from BioRender (https://www.biorender.com)

The mouse (C57BL/6) brain-derived BV-2 microglial cell line (Code No. 0356) was purchased from the Rio de Janeiro Cell Bank (BCRJ, Rio de Janeiro, RJ, Brazil). BV-2 cells were cultured using *Roswell Park Memorial Institute* (RPMI) 1640 medium containing antibiotics (1% penicillin/streptomycin, 10.000 U/mL; 10 mg/mL, respectively) and supplemented with fetal bovine serum (FBS, 10%). The cells were maintained following standard conditions using a humidified and controlled atmosphere of carbon dioxide (CO_2_, 5%) at 37 °C. The medium was renewed at least 4 times per week until a proper number of cells was obtained to proceed with the experiments [62]. For the experiments, BV-2 cells were seeded in different multi-well plates, including 96-well plates at a density of 1 × 10^5^ cells/well and 6-well plates at 5 × 10^5^ cells/well. Initially, BV-2 cells were treated with an increasing concentration-effect curve of glucose (GL) and CA, consisting of 0.5, 1, 5, 10, 50, 75, 100, 200, and 300 mM or µM, respectively (Fig. 1). The morphology of cells was observed under optical microscopy during the exposure period and throughout the course of the treatments (Fig. S1).

In order to characterize the effects of exposure to high glucose and the treatment with CA, and based on the initial concentration-effect curves, BV-2 microglial cells were divided into different groups:

(I) The control group (C; untreated cells) consisted of cells that were exposed only to the culture medium for 48 h. This group represents the normoglycemic state, with a glucose concentration of 5 mM. This group did not receive glucose or CA treatments.
(II) The CA control groups were pre-exposed only to the culture medium for 24 h and then treated only with CA at the concentrations of 50 µM and 75 µM for another period of 24 h. These groups did not receive glucose treatment.
(III) The glucose (GL) group was treated for 48 h at the concentration of 25 mM, which was used as a positive hyperglycemic control, since studies have suggested that this concentration would mimic the human glycemic levels in the diabetic state (450 mg/dL) [9, 23, 63, 64]. This group did not receive CA treatment.
(IV) The groups exposed to the association of GL and CA (50 µM and 75 µM) treatments were pre-exposed to high glucose (25 mM) for a 24-h period. Subsequently, CA (50 µM and 75 µM) treatments were added and left for 24 h without removing GL, totalizing 48 h of exposure to high glucose (Fig. 1).

Additionally, a concentration-effect curve of mannitol (osmotic control, neither taken up nor metabolized by the cells) ranging from 5 to 100 mM for 24 h and 48 h was also performed to investigate possible effects of media osmolality (Fig. S2) [65–67].

### MTT Assay

Cellular viability and proliferation were measured through the MTT (3-(4,5-Dimethyl-2-thiazolyl)−2,5-diphenyl-2 H-tetrazolium bromide) assay [68]. MTT, a yellow salt, is metabolized by mitochondrial dehydrogenase enzymes to a violet compound (formazan), which can be quantified spectrophotometrically. Firstly, cells were seeded in 96-well plates at a density of 1 × 10^5^ cells/well and treated with the glucose and CA curves for 24 h and 48 h. Following the incubation period, cells were gently washed with phosphate-buffered saline (PBS 0.01 M, pH 7.4) to remove the treatments and avoid interference. Subsequently, cells were incubated with the MTT reagent (5 mg/mL, dissolved in PBS). After 4 h of incubation at 37 °C, the reagent was removed. Dimethylsulfoxide (DMSO) was added to solubilize formazan crystals and the absorbance was measured at 570 nm. The results were expressed as percentages in relation to the control group.

### Oxidative Metabolism Indicators

#### Nitric Oxide (NO) Levels

The determination of nitric oxide (NO) levels was performed indirectly by measuring the nitrite levels, using the Griess reagent [69]. Briefly, the cell supernatant was incubated with the Griess reagent for 15 min at room temperature, avoiding light exposure. Absorbance was measured at 540 nm, and the results were expressed as percentages in relation to the control group.

#### TBARS Assay

Lipid peroxidation was determined in samples in accordance with the established methodology [70]. Reactive species react with lipids, resulting in the release of malondialdehyde (MDA), which then reacts with thiobarbituric acid (TBA) to form a pink-colored product. Briefly, the samples were incubated in a water bath at 95 °C in a medium comprising sodium dodecyl sulfate (8.1%), acetate buffer (20%), and TBA (0.8%). The absorbance was measured at 532 nm and the results were expressed as percentages in relation to the control group and as nmol MDA/mg protein.

#### Reactive Species Production

Reactive species (RS) formation was determined in cell cultures using the DCFH-DA reagent [71]. The DCFH-DA reagent diffuses across cell membranes and is subsequently deacetylated by intracellular esterases, resulting in the formation of 2’,7’-dichlorodihydrofluorescein (DCFH). In the cytosol, DCFH reacts with RS, generating a fluorescent product (2’−7’-dichlorofluorescein, DCF). Briefly, the samples were incubated with DCFH-DA for 1 h at room temperature, while ensuring protection from light. The oxidation of DCFH-DA to DCF was recorded at 488 nm excitation and at 525 emission. Results were expressed as U DCF/mg protein.

### Purinergic System Enzymes Activities

#### NTPDase and E-5′-Nucleotidase Activity Assays

The activities of E-NTPDase (using ATP and ADP as substrates) [72] and E-5′-NT (using AMP as substrate) [73] were determined by the release of inorganic phosphate (Pi). The reaction system for E-NTPDase comprised the following components: 50 mM KCl, 15 mM CaCl_2_, 1 mM EDTA, 100 mM glucose, 2250 mM sucrose, and 450 mM Tris-HCl buffer (pH 7.4). The reaction system for E-5′-NT consisted of 100 mM MgSO_2_ and 1000 mM Tris-HCl buffer (pH 7.0). Following completion of the treatments under adherent culture conditions, the culture medium was removed, and the cells were gently detached from the plates. The resulting cell suspension was resuspended in saline (NaCl, 0.9%), and 20 µL were added to the reaction system of each enzyme and preincubated at 37 °C for 10 min. The enzymatic reaction was initiated by adding the specific substrates for each enzyme: ATP and ADP (1 mM) for E-NTPDase, and AMP (2 mM) for E-5’-NT. Following a 30-min incubation period, the reactions were stopped by the addition of TCA (10%). The inorganic phosphate released as a result of nucleotide hydrolysis was then quantified using malachite green as the colorimetric reagent. A standard curve was prepared with KH_2_PO_4_ and used to determine the released Pi. The absorbance was then measured at a wavelength of 630 nm. Results were expressed as nmol Pi/min/mg of protein.

#### Adenosine Deaminase (ADA) Activity Assay

ADA activity was measured indirectly through the ammonia production [74]. This method consists in the quantification of a colored indophenol complex derived from ammonia, produced when ADA acts in excess of Ado. Cell suspensions prepared as described above were used for ADA activity determination. Briefly, 50 µL of each cell suspension was incubated with 21 mmol/L adenosine (pH 6.5) at 37 °C for 60 min. After the incubation, the reaction was stopped by the addition of 167.8 mM sodium nitroprusside, 106.2 mM phenol, and a sodium hypochlorite solution. Ammonium sulfate (75 mM) was used as a standard. Controls were performed for each sample to correct for any ammonia present in the sample. The amount of ammonia was quantified at 620 nm and the results were expressed as U Ado/mg of protein.

### Western Blot

The density of purinoceptors (P2X7R, A1R, and A2AR), inflammatory markers (NLRP3 inflammasome and IL-1β interleukin), and microglial activation marker (ionized calcium-binding adapter molecule 1, IBA-1), were assessed by Western blot [75]. Cells were initially homogenized in ice-cold radioimmunoprecipitation assay (RIPA) buffer, which was supplemented with 1 mM protease and phosphatase inhibitors. The resulting suspension was then centrifuged for 12,000 rpm at 4 °C for 10 min. The protein concentration was determined using the Lowry method. Subsequently, samples were separated using sodium dodecyl sulfate-polyacrylamide gel electrophoresis (SDS-PAGE, 10%) and transferred to nitrocellulose membranes (Bio-Rad Laboratories, Hercules, CA, USA). Subsequently, membranes were blocked with 5% bovine serum albumin (BSA) in 0.1% TBS-T for 1 h and then incubated overnight at 4 °C with the primary antibodies: P2X7R (1:500; Santa Cruz Biotechnology, CA, USA), A1R (1:500; Thermo Fisher Scientific), A2AR (1:1000; Thermo Fisher Scientific), NLRP3 (1:1000; Thermo Fisher Scientific), IL-1β (1:500; CUSABIO, Hubei, China), and IBA-1 (1:1000; Thermo Fisher Scientific), followed by incubation with appropriate secondary antibodies (1:10,000; Thermo Fisher Scientific) for 90 min at room temperature. Finally, membranes were incubated with an enhanced chemifluorescent substrate (Immobilon^®^ Forte Western HRP Substrate, Merck KGaA) and analyzed using an Amersham Imager 600 (GE, Healthcare Life Sciences, EUA) equipment. Membranes were reprobed and tested for β-actin immunoreactivity (1:1000, Santa Cruz Biotechnology) as a control for protein concentration.

### Apoptosis and Cell Death Assay

Apoptosis and cell death were assessed by flow cytometry analysis using the FITC Annexin V and Propidium Iodide (PI) staining kit (BD Pharmingen™, BD Biosciences) following the manufacturer’s instructions. In this technique, FITC Annexin V probe discriminates cells undergoing apoptosis, whereas PI dye stains dead cells. Following the seeding and treatments of BV-2 cells, cell samples were harvested from plates and freshly prepared for analysis using a FACSCanto™ II Flow Cytometer (BD Biosciences, San Jose, CA). Data were then analyzed using the FlowJo vX 10.6 software. The following criteria were adopted for the classification of cell populations in samples: (a) lower left: viable cells, both FITC Annexin V and PI negative; (b) lower right: cells undergoing early apoptosis, FITC Annexin V positive and PI negative; (c) upper left: necrotic cells, FITC Annexin V negative and PI positive; (d) upper right: cells in the final stages of apoptosis and dead cells, both FITC Annexin V and PI positive. The results are reported as percentages of viable, apoptotic, or dead cells [76].

### Cell Cycle Analysis

Cell cycle analysis of BV-2 cells at a density of 2 × 10^5^ cells/well exposed to glucose (25 mM), and treated with CA selected concentrations was performed similarly to previously described [77]. For this protocol, cells were initially washed with PBS and resuspended in ethanol 70%. Then, cells were vortexed and kept at 4 °C overnight, centrifuged, and washed again with PBS. Thereafter, cells were stained with 500 µL of the staining solution diluted in PBS (50 µg/mL PI, 100 µg/mL RNase-free DNase, and 0.05% Triton X-100) and incubated for 40 min at 37 °C. Following this step, cells were washed once more with PBS and finally resuspended in 500 µL of PBS for cell cycle phases determination by flow cytometry using a FACSCanto™ II Flow Cytometer. Data acquired by the equipment were analyzed using the FlowJo vX. 10.6 software.

### Protein Determination

The protein content of the samples was determined using the Coomassie Blue reagent and BSA as a standard [78]. In brief, 10 uL of each sample was added to a 96-well plate and incubated with the Coomassie Blue reagent for 10 min in the dark at room temperature. Then, absorbance was read at 595 nm and the values were interpolated against a standard BSA curve (0.1–2.0 mg/mL). The protein of the samples (mg/mL) was adjusted to each protocol requirement.

### Statistical Analysis

The data obtained from the experiments were initially plotted in Microsoft Excel (Microsoft Office Professional Plus 2019 Version) and subsequently converted to percentages relative to the control group (untreated cells). The Shapiro-Wilk test was used to assess the normality of data. Data were analyzed by one or two-way ANOVA followed by Bonferroni’s post hoc test using the GraphPad Prism v.9 statistical package (GraphPad Software, Inc.; La Jolla, CA, USA). Statistically significant differences between groups were considered when *p* < 0.05. All data were expressed as mean ± standard error of the mean (SEM) of at least three independent experiments.

## Results

### High Glucose Affects Cell Viability, Proliferation, and NO Production of Microglial Cells

**Fig. 2:**
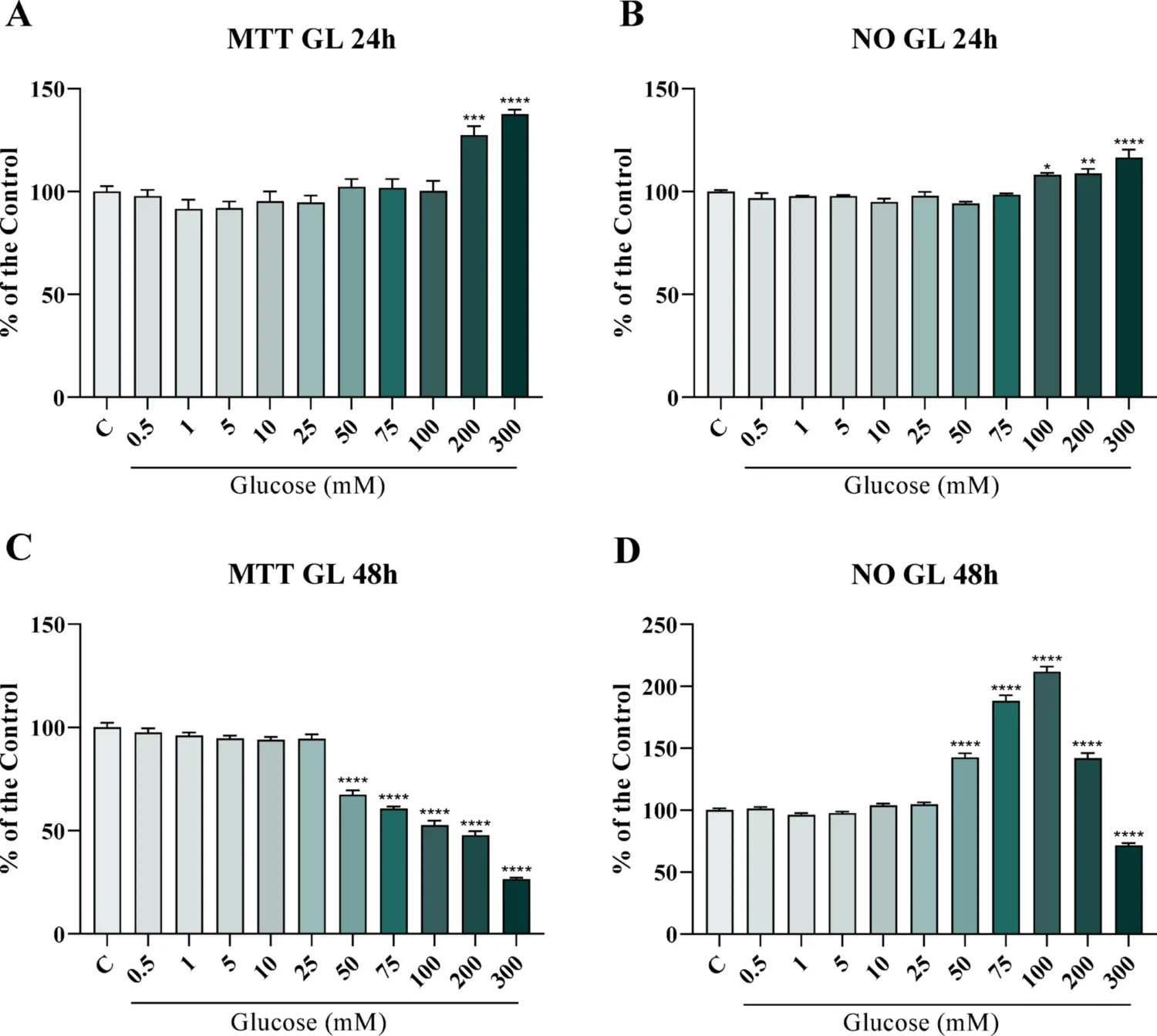
In vitro effects of different concentrations of GL (0.5–300 mM) in **A**, **C** cell viability, and **B**, **D** nitric oxide levels (NO) of BV-2 microglial cells after 24 h and 48 h of exposure. Cell viability and proliferation was assessed by the MTT assay. NO was assessed indirectly by nitrite levels using the Griess reagent. C = Control group; GL = Glucose. Results are expressed as a percentage of the control group. Bars represent means ± SEM. One-way ANOVA was followed by the Bonferroni’s post-test. Significant values are considered at *P* ≤ 0.05, *n* = 8. *indicates *P* ≤ 0.05; **P ≤ 0.01; ***P ≤ 0.001; ****P ≤ 0.0001

Initially, to evaluate the effects of high glucose concentrations in vitro, which mimic hyperglycemia found in the diabetic state, on the viability of microglial cells, we exposed BV-2 cells to a concentration curve of glucose (0.5–300 mM) over a 24 h and 48 h period. The results showed that after 24 h, the glucose concentrations of 200 and 300 mM (Fig. 2A) significantly increased cell viability compared to untreated control cells. Furthermore, our findings revealed that after 48 h glucose concentrations up to 25 mM did not affect the viability of BV-2 cells. However, glucose concentrations of 50–300 mM exhibited a notable reduction in cell viability compared to the control group (Fig. 2C). Additionally, findings from the osmotic control (mannitol) curve did not show significant alterations in cell viability after 24 h and 48 h (Fig. S2).

Moreover, glucose concentrations of 100, 200, and 300 mM, after 24 h of exposure (Fig. 2B) and concentrations of 50–200 mM after 48 h (Fig. 2D), increased NO production in microglial cells when compared to untreated control cells. The concentration of 300 mM decreased NO levels compared to the control group (Fig. 2D). Based on these findings, the glucose concentrations of 10, 25, and 50 mM and the 48-h treatment period were selected for the subsequent cytotoxicity assay (Fig. S3 and Fig. S4), to choose a single glucose concentration for the next experiments.

### Cytotoxicity and Oxidative Metabolism Indicators of CA-Treated BV-2 Cells

**Fig. 3:**
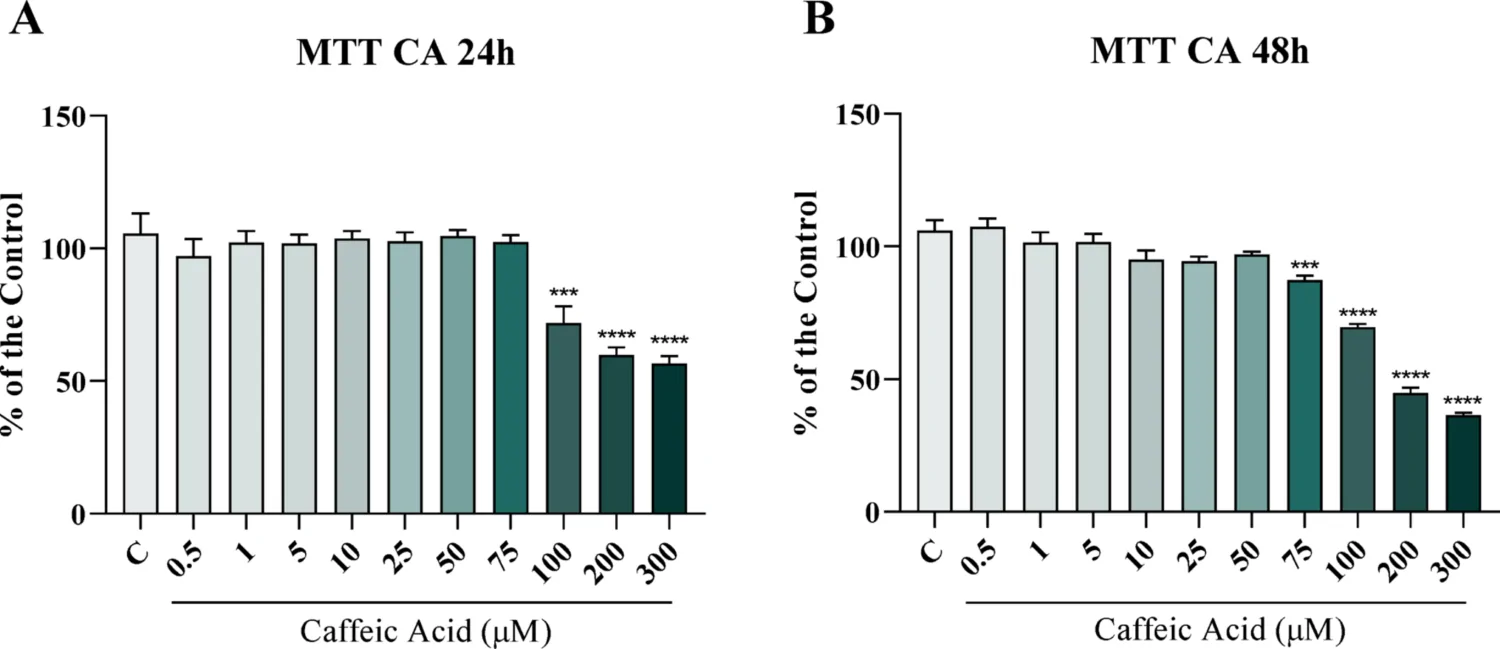
In vitro effects of different concentrations of CA (0.5–300 µM) on cell viability of BV-2 microglial cells. Cell viability and proliferation was assessed by the MTT assay. **A** After 24 h of treatment, **B** After 48 h of treatment. C = Control group; CA = Caffeic Acid. Results are expressed as a percentage of the control group. Bars represent means ± SEM. One-way ANOVA was followed by the Bonferroni’s post-test. Significant values are considered at *P* ≤ 0.05, *n* = 8. *indicates *P* ≤ 0.05; **P ≤ 0.01; ***P ≤ 0.001; ****P ≤ 0.0001

To determine whether the CA concentrations utilized in our study did not have toxicity to microglial cells, we evaluated the effects of CA per se on the viability and proliferation of BV-2 cells. Cells were incubated with a concentration-effect curve of CA (0.5–300 µM) for 24 and 48 h. Figure 3A shows that after 24 h of treatment, CA concentrations up to 75 µM did not alter the viability of microglial cells, whereas CA concentrations of 100–300 µM significantly decreased cell viability compared to untreated control cells. Moreover, after 48 h of treatment, concentrations ranging from 75 to 300 µM of this phenolic acid revealed a notorious cytotoxicity compared to the control group of cells (Fig. 3B).

**Fig. 4:**
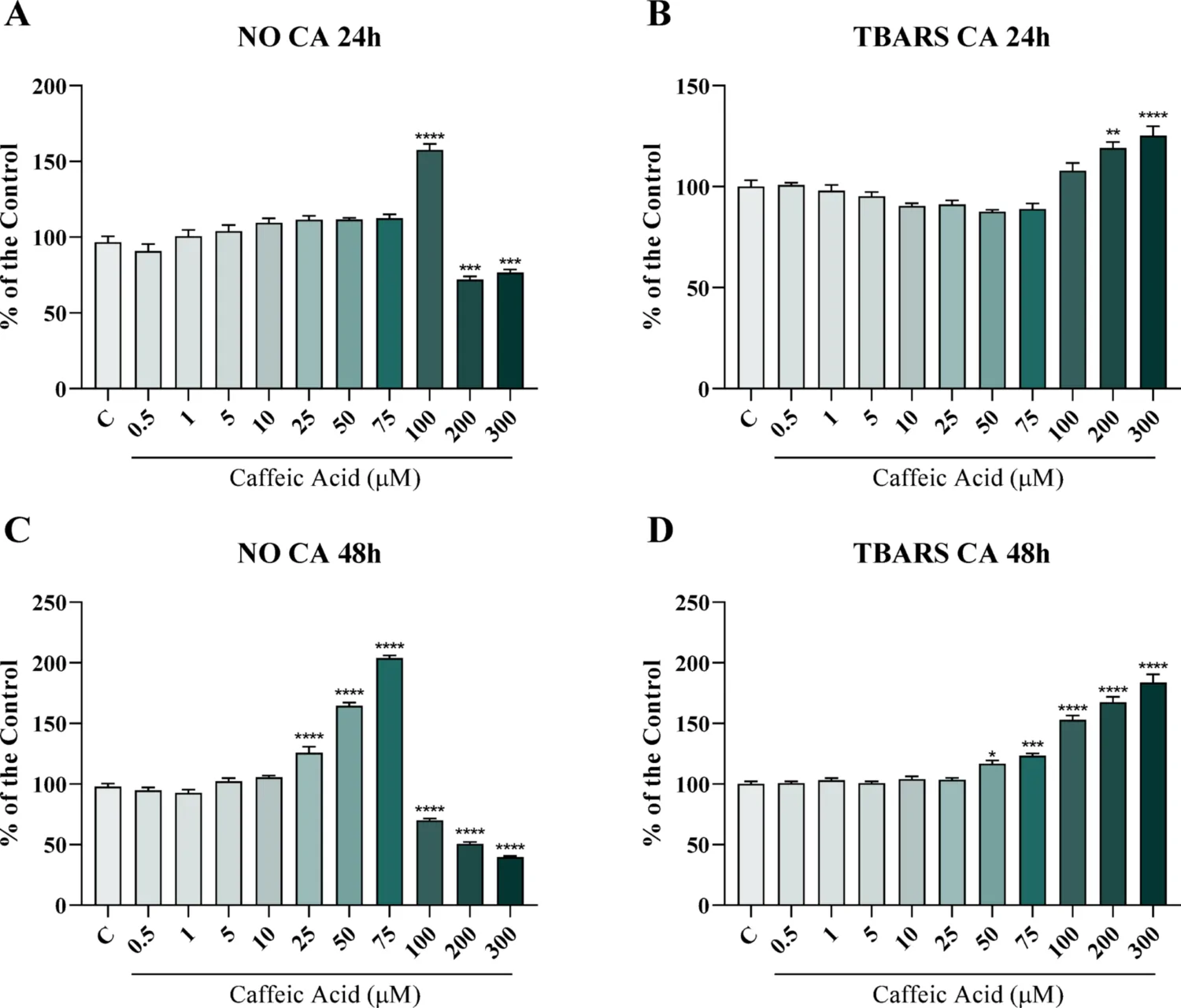
In vitro effects of different concentrations of CA (0.5–300 µM) on oxidative parameters of BV-2 microglial cells.** A**,** C** Nitric oxide levels (NO) and **B**,** D** Thiobarbituric acid reactive substances (TBARS) levels after 24 h and 48 h of treatment. C = Control group; CA = Caffeic Acid. Results are expressed as a percentage of the control group. Bars represent means ± SEM. One-way ANOVA was followed by the Bonferroni’s post-test. Significant values are considered at *P* ≤ 0.05, *n* = 8. *indicates *P* ≤ 0.05; **P ≤ 0.01; ***P ≤ 0.001; ****P ≤ 0.0001

Additionally, we examined oxidative metabolism markers to assess whether CA could be altering the production of NO and TBARS levels, which could potentially induce cellular damage. Consequently, the NO levels and lipid peroxidation (assessed by TBARS levels) of BV-2 cells were evaluated over a 24- and 48-h period using the same concentration curve for CA. Figure 4A illustrates that after 24 h, the treatment with CA at 100 µM increased NO levels, whereas concentrations of 200 and 300 µM decreased this parameter compared to untreated control cells. CA concentrations of 200 and 300 µM also increased the lipid peroxidation after 24 h compared to the control group (Fig. 4B).

Also, CA concentrations of 25, 50 and 75 µM increased NO levels after 48 h, whereas concentrations of 100–300 µM decreased NO levels compared to control cells (Fig. 4C). CA concentrations from 50 to 300 µM increased TBARS levels after 48 h compared to the control group of microglial cells (Fig. 4D). Therefore, based on the results of cell viability and oxidative metabolism indicators, two concentrations of CA, 50 and 75 µM, and the 24 h treatment period were chosen for further assays.

### Oxidative Metabolism Indicators of Microglial Cells Challenged with High Glucose and Treated with CA

In light of prior research indicating that elevated glucose levels in microglial cell cultures promote microglial activation and the generation of free radicals [17, 20, 79], we evaluated the effects of CA on RS production and lipid peroxidation in BV-2 cells exposed to a high glucose concentration.

**Fig. 5:**
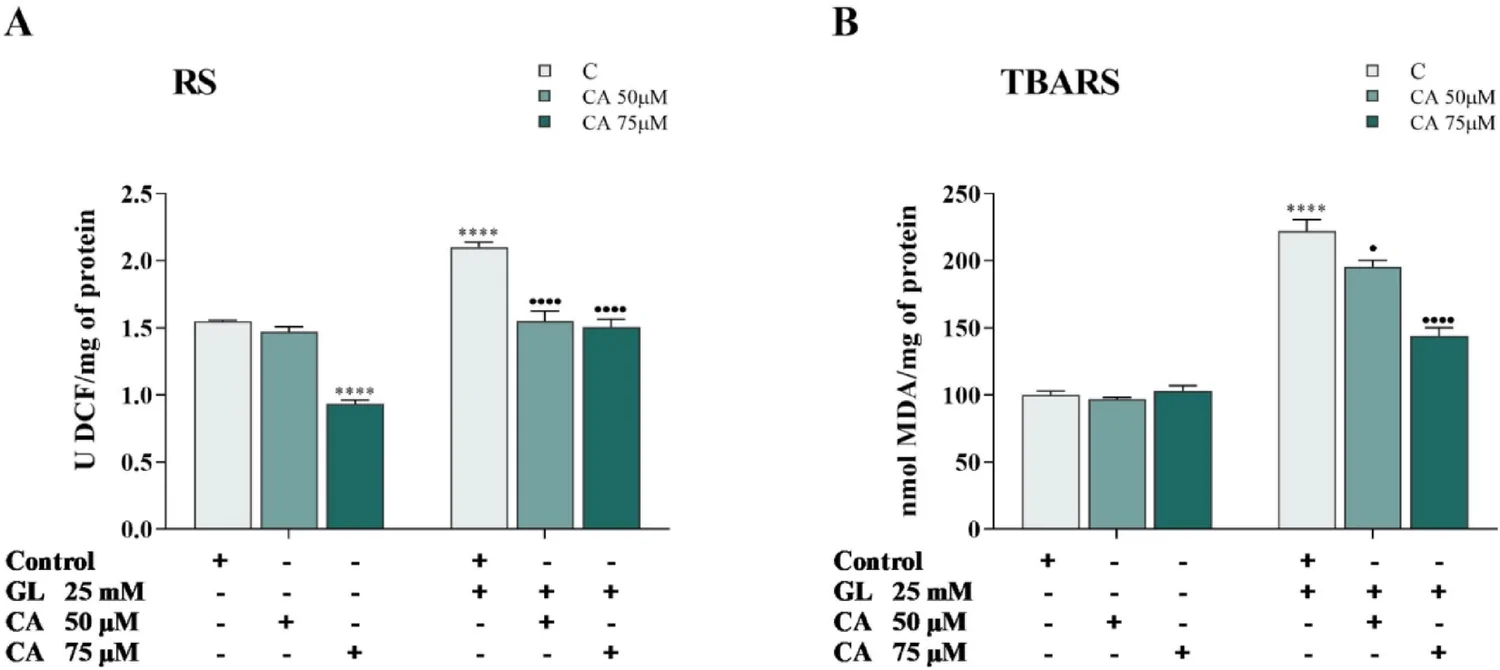
Oxidative parameters of BV-2 microglial cells exposed in vitro to a high glucose (25 mM) medium for 48 h and treated with CA (50 and 75 µM) for 24 h.** A** Reactive species (RS) levels, and **B** Thiobarbituric acid reactive substances (TBARS) levels. Control = Control group; GL = High glucose group; and CA = Caffeic Acid. Results are expressed as U DCF/mg of protein (**A**) and nmol MDA/mg protein (**B**). Bars represent means ± SEM. Two-way ANOVA was followed by the Bonferroni’s post-test. Significant values are considered at *P* ≤ 0.05, *n* = 6. (*) Indicates a significant difference in relation to the control group, (•) indicates a significant difference in relation to the high glucose group. *P ≤ 0.05; **P ≤ 0.01; ***P ≤ 0.001; ****P ≤ 0.0001

The effects of CA treatment on RS levels are shown in Fig. 5A. Exposure to the selected high glucose (25 mM) concentration significantly increased RS levels by approximately 36% compared to the untreated control group. Interestingly, treatment with CA per se at 75 µM reduced RS levels by approximately 40% compared to the untreated control group. In cells exposed to high glucose, treatment with CA at both 50 and 75 µM significantly attenuated the increase in RS levels (26% and 28%, respectively) compared to the untreated high glucose group.

We next investigated whether CA could attenuate lipid peroxidation in BV-2 cells, as assessed by TBARS levels (Fig. 5B). Exposure to high glucose significantly increased TBARS levels compared to the untreated control group, indicating enhanced lipid peroxidation. Treatment with CA at 50 and 75 µM significantly reduced TBARS levels in high glucose-challenged cells compared to the untreated high glucose group, with reductions of approximately 11% and 35%, respectively.

### CA Modulates the Activity of Purinergic Ectoenzymes

**Fig. 6:**
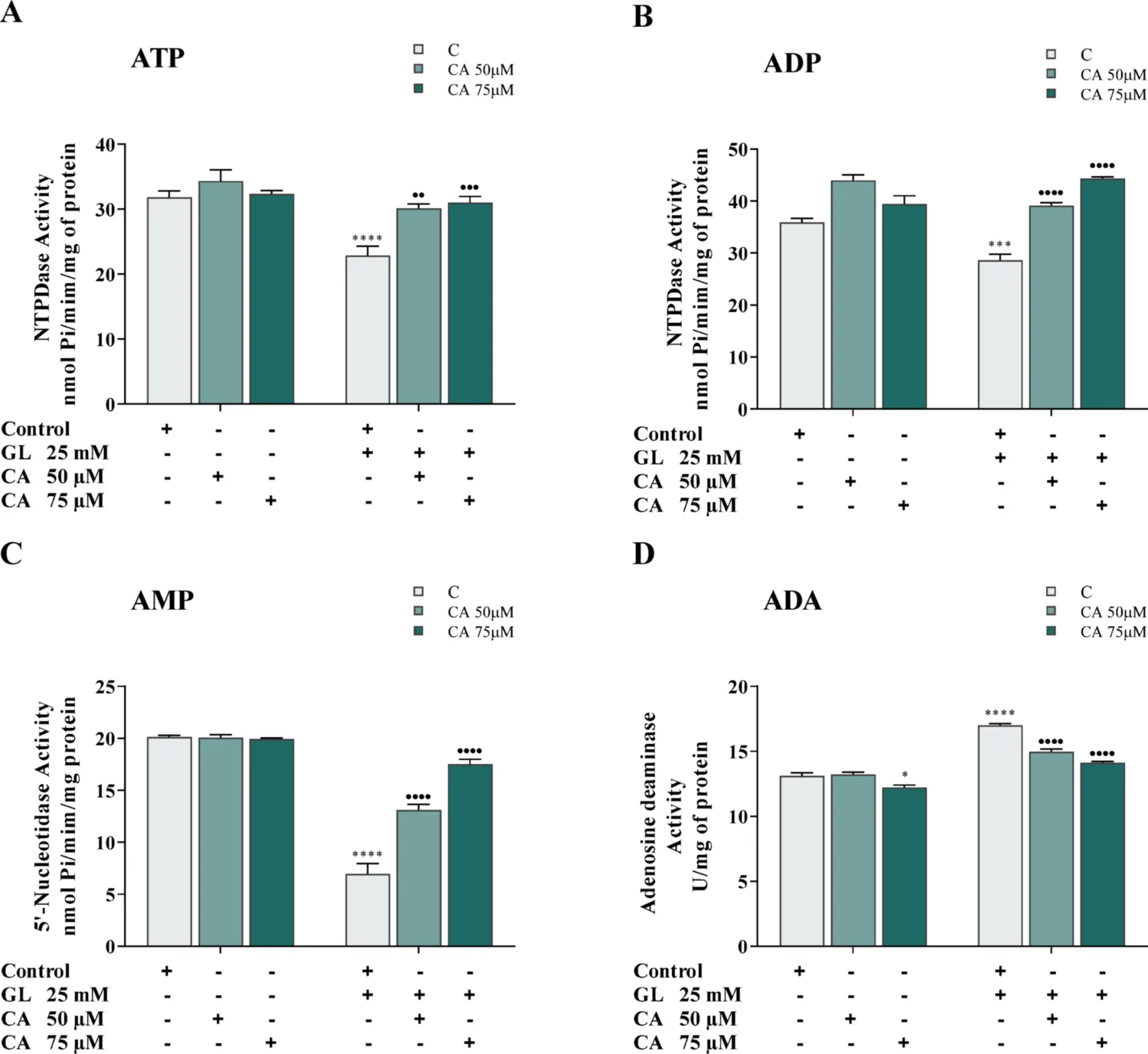
Purinergic enzyme activities of BV-2 microglial cells exposed in vitro to a high glucose (25 mM) medium for 48 h and treated with CA (50 and 75 µM) for 24 h.** A** NTPDase activity using ATP, and **B** ADP as substrates, **C** 5’-NT activity using AMP as substrate, and **D** ADA activity using adenosine (Ado) as substrate. C = Control group; CA = Caffeic Acid group, and GL = High glucose group. Results are expressed as nmol Pi/min/mg protein (**A**–**C**) and U/mg protein (**D**). Bars represent means ± SEM. Two-way ANOVA was followed by the Bonferroni’s post-test. Significant values are considered at *P* ≤ 0.05, *n* = 6. (*) Indicates a significant difference in relation to the control group, (•) indicates a significant difference in relation to the high glucose group. *P ≤ 0.05; **P ≤ 0.01; ***P ≤ 0.001; ****P ≤ 0.0001

Figure 6 shows the effects of CA (50 and 75 µM) on E-NTPDase, E-5’-NT, and ADA activities in BV-2 microglial cells exposed to high glucose (25 mM). The findings demonstrated a reduction in E-NTPDase activity regarding ATP (28%, Fig. 6A) and ADP (20%, Fig. 6B) hydrolysis in the high glucose group when compared to the untreated control group. Additionally, the results indicated that CA at the concentrations of 50 and 75 µM increased ATP (32% and 36%, respectively, Fig. 6A) and ADP (37% and 55%, respectively, Fig. 6B) hydrolysis in BV-2 cells exposed to high glucose and treated with CA when compared to the untreated high glucose group.

The activity of E-5’-NT was then evaluated through the assessment of AMP hydrolysis. The findings showed that exposure to high glucose decreased (65%, Fig. 6C) AMP hydrolysis when compared to untreated control cells. However, treatment with CA (50 and 75 µM) significantly increased hydrolysis of this nucleotide (88% and 151%, respectively) in high glucose-exposed microglial cells when compared to the untreated high glucose group.

Adenosine breakdown was investigated as an indicator of ADA activity (Fig. 6D). A decrease per se of 7% in ADA activity was observed in the control group treated with CA at 75 µM when compared to untreated control cells. Moreover, ADA activity was increased by 30% in high glucose-exposed cells when compared to the untreated control group. In addition, treatment with CA (50 and 75 µM) decreased ADA activity (12% and 17%, respectively) in cells exposed to high glucose compared to the untreated high glucose group.

Overall, the treatment with CA (50 and 75 µM) in BV-2 microglial cells exposed to high glucose was able to restore, at least partially, ATP, ADP, and AMP hydrolysis, and Ado breakdown. Based on these results, we can propose that CA and high concentrations of glucose significantly promote changes in the metabolism of these molecules, indirectly assessed by enzymatic activities.

### Effect of CA on the Density of Purinergic Receptors in BV-2 Microglial Cells Exposed to High Glucose

Considering previously published results on the effects of CA on the density of purinoceptors [59] and since CA modulated ectonucleotidase enzyme activities in our study, we also investigated whether this phenolic compound could alter the density of the purinergic receptors P2X7R, A1R, and A2AR in vitro in BV-2 microglial cells exposed to high glucose.

**Fig. 7:**
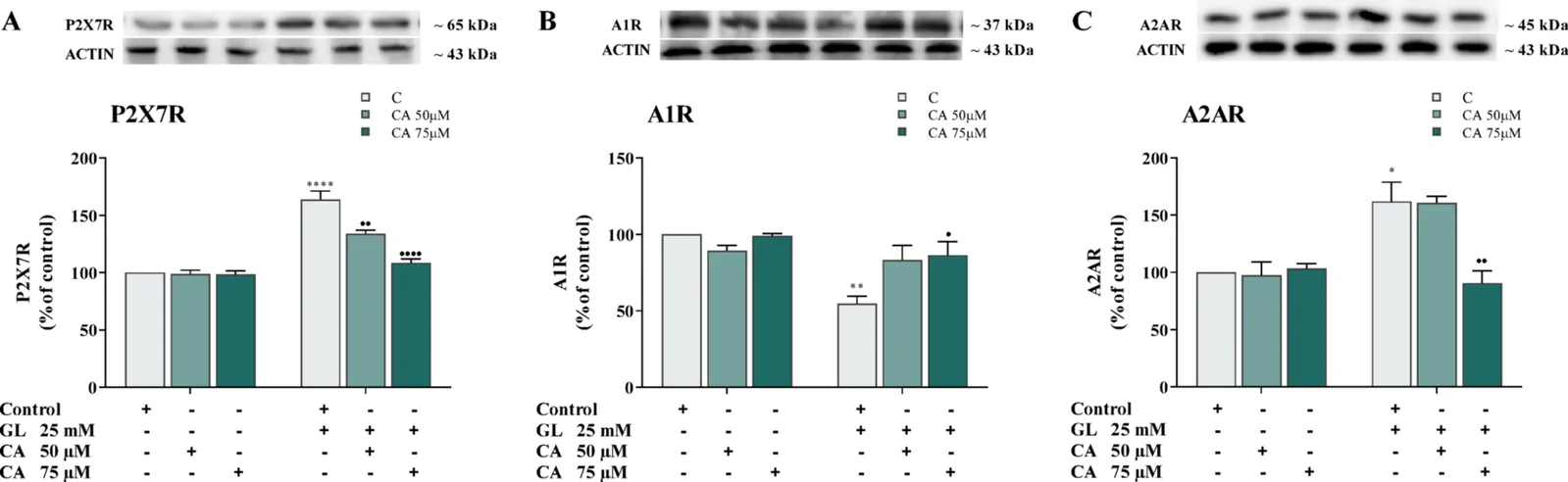
Western blot analysis of the density of purinoceptors in BV-2 microglial cells exposed in vitro to high glucose (25 mM) for 48 h and treated with CA (50 and 75 µM) for 24 h.** A** P2X7R, **B** A1R, and **C** A2AR. β-actin was used as a loading control to normalize protein levels. C = Control group; CA = Caffeic Acid group, and GL = High glucose group. Bars represent means ± SEM. Two-way ANOVA was followed by the Bonferroni’s post-test. Significant values are considered at *P* ≤ 0.05, *n* = 3. (*) Indicates a significant difference in relation to the control group, (•) indicates a significant difference in relation to the high glucose group. *P ≤ 0.05; **P ≤ 0.01; ***P ≤ 0.001; ****P ≤ 0.0001

Our results showed an increase in P2X7R (64%, Fig. 7A) and A2AR (62%, Fig. 7C) densities in cells exposed to high glucose when compared to untreated control cells. CA treatment (50 and 75 µM) decreased P2X7R (18% and 34%, respectively, Fig. 7A) density in microglial cells exposed to high glucose when compared to untreated high glucose-challenged cells. Nevertheless, only the CA treatment at 75 µM managed to decrease A2AR density (44%, Fig. 7C) in high glucose cells treated with CA when compared to the high glucose untreated cells. Regarding A1R density, high glucose-exposed cells showed significantly decreased density of this receptor (45%, Fig. 7B) compared to untreated control cells. Only the high glucose group that was treated with CA at 75 µM showed an increase (58%, Fig. 7B) in A1R receptor density compared to the untreated high glucose group.

In brief, Western blot analysis showed that CA was capable of modulating the protein density of purinergic receptors by downregulating the density of the P2X7R and A2AR purinoceptors and upregulating the density of the A1R purinoceptor compared to untreated and high glucose-exposed cells.

### CA Attenuates Inflammatory Responses in BV-2 Microglial Cells Exposed to High Glucose

**Fig. 8:**
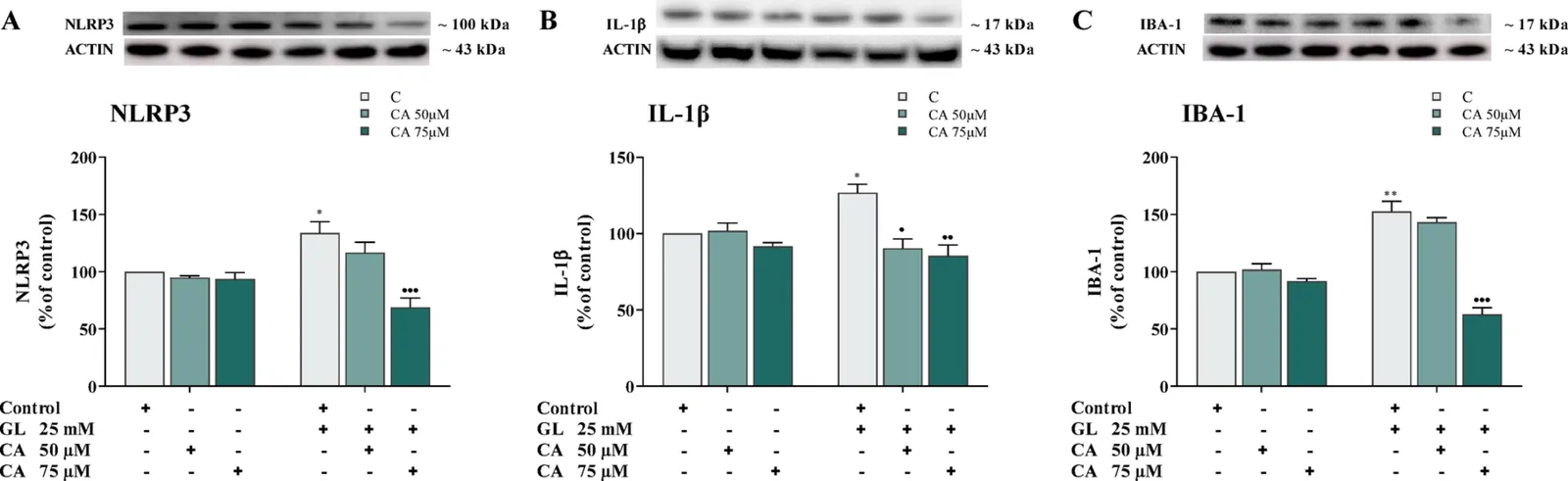
Western blot analysis of the density of inflammatory parameters in BV-2 microglial cells exposed in vitro to high glucose (25 mM) for 48 h and treated with CA (50 and 75 µM) for 24 h.** A** NLRP3 inflammasome, **B** Interleukin IL-1β, and **C** IBA-1. β-actin was used as a loading control to normalize protein levels. C = Control group; CA = Caffeic Acid group, and GL = High glucose group. Bars represent means ± SEM. Two-way ANOVA was followed by the Bonferroni’s post-test. Significant values are considered at *P* ≤ 0.05, *n* = 4. (*) Indicates a significant difference in relation to the control group, (•) indicates a significant difference in relation to the high glucose group. *P ≤ 0.05; **P ≤ 0.01; ***P ≤ 0.001; ****P ≤ 0.0001

Figure 8 shows the protein density of the NLRP3 inflammasome, IL-1β, and IBA-1. Compared to the untreated control group, exposure to high glucose significantly increased the density of NLRP3, IL-1β, and IBA-1 by 34% (Fig. 8A), 27% (Fig. 8B), and 53% (Fig. 8C), respectively. Regarding NLRP3 density, treatment with CA at 75 µM significantly reduced this parameter by 48% in high glucose-exposed cells compared to the high glucose group (Fig. 8A). Similarly, treatment with CA at 50 and 75 µM significantly reduced IL-1β density by 29% and 33%, respectively, compared to the untreated high glucose group (Fig. 8B). Furthermore, CA treatment at 75 µM reduced IBA-1 density by 58% compared to high glucose-challenged and untreated cells (Fig. 8C).

### CA Attenuated Cell Death Induced by High Glucose and Modulated the Cell Cycle Phases of Microglial Cells

**Fig. 9:**
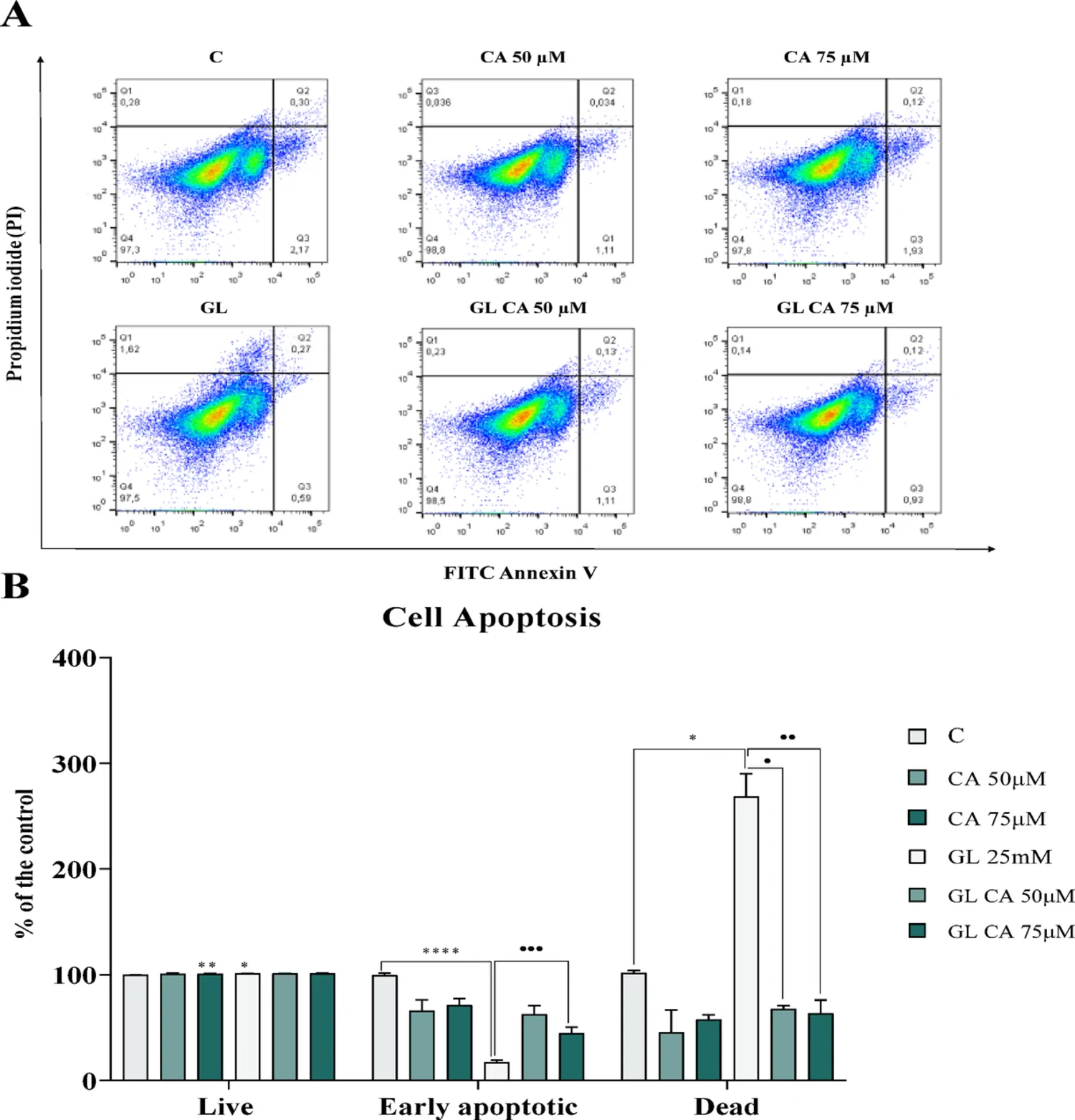
Apoptosis by flow cytometry analysis of BV-2 microglial cells exposed in vitro to high glucose (25 mM) for 48 h and treated with CA (50 and 75 µM) for 24 h.** A** The representative dot-plot profile of apoptosis and cell death was measured by using the FITC Annexin V apoptosis probe and the PI dye. Viable cells were identified as FITC Annexin V−/PI− (lower left); Early apoptotic cells were considered FITC Annexin V+/PI− (lower right); Late apoptotic/dead cells were considered as FITC Annexin V+/PI+ (upper left and right). C = Control group; CA = Caffeic Acid group, and GL = High glucose group. **B** Quantitative analysis of the parameters: live, early apoptotic, and dead cells from (**A**). Results are expressed as a percentage of the control group. Bars represent means ± SEM. Two-way ANOVA was followed by the Bonferroni’s post-test. Significant values are considered at *P* ≤ 0.05, *n* = 6. (*) indicates a significant difference in relation to the control group of each parameter, (•) indicates a significant difference in relation to the high glucose group of each parameter. *P ≤ 0.05; **P ≤ 0.01; ***P ≤ 0.001; ****P ≤ 0.0001

Considering the cytotoxic effects of high glucose concentrations in microglial cells, we further investigated the induction of apoptosis/cell death and modulation of the cell cycle in BV-2 cells in a high glucose medium (25 mM) and treatment with CA (50 and 75 µM). Apoptosis and cell death analysis was conducted using flow cytometry with the FITC Annexin V and propidium iodide (PI) kit. Particularly, Fig. 9 shows that exposure to high glucose (GL, 25 mM) significantly increased (165%) the number of cells in advanced stages of apoptosis or already dead, which were represented here as dead cells, when compared to cells of the untreated control group. On the other hand, treatment with CA 50 and 75 µM (75% and 76%, respectively) decreased the number of dead cells induced by the high glucose medium (Fig. 9B).

**Fig. 10:**
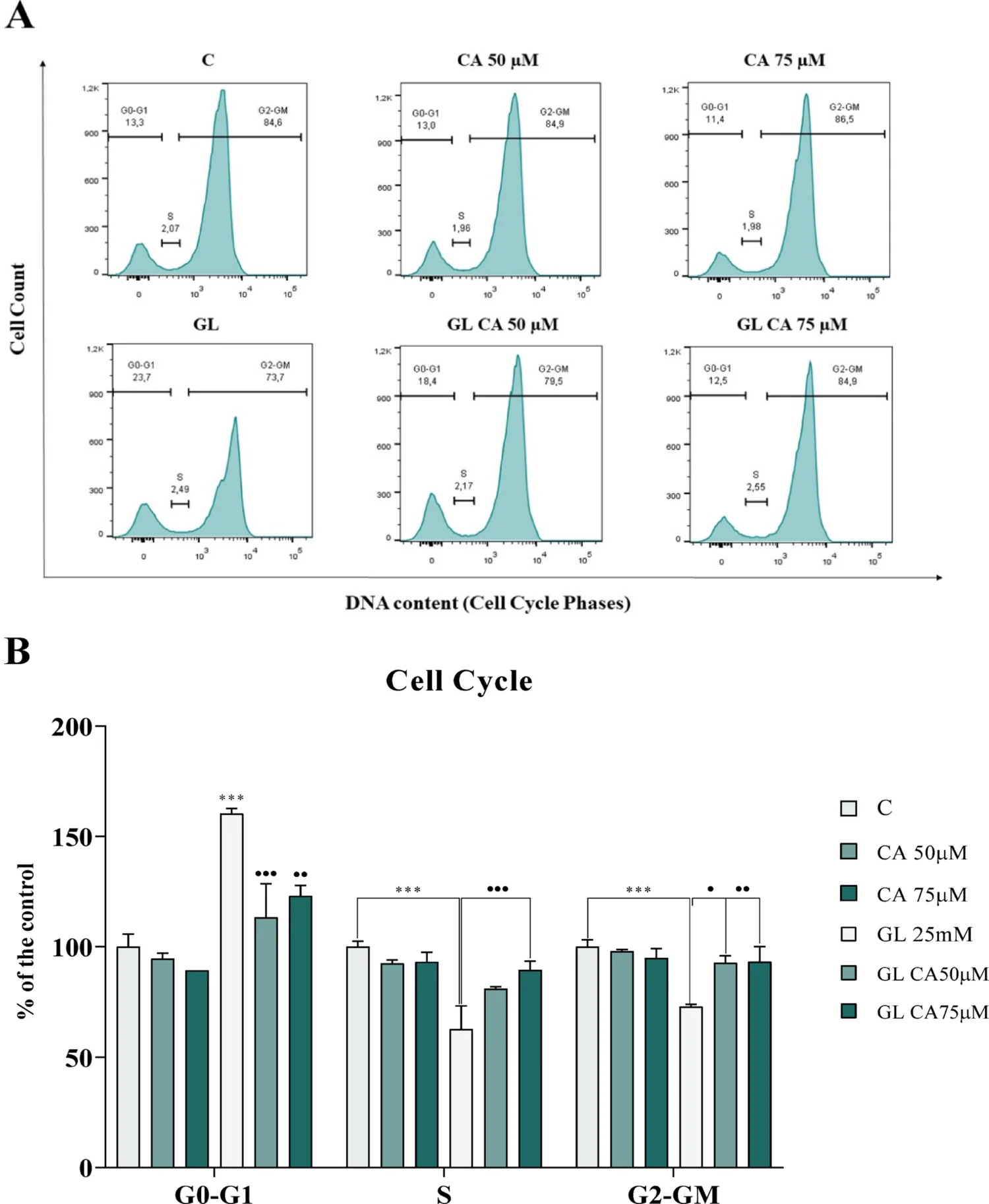
Cell cycle profile by flow cytometry of BV-2 microglial cells exposed in vitro to high glucose (25 mM) for 48 h and treated with CA (50 and 75 µM) for 24 h.** A** Representative histograms, and **B** Graphs showing the DNA content in each phase of the cell cycle for each group. Cell cycle parameters: G0/G1, S, and G2/M. C = Control group; CA = Caffeic Acid group, and GL = High glucose group. Results are expressed as a percentage of the control group. Bars represent means ± SEM. Two-way ANOVA was followed by the Bonferroni’s post-test. Significant values are considered at *P* ≤ 0.05, *n* = 3. (*) indicates a significant difference in relation to the control group of each parameter, (•) indicates a significant difference in relation to the high glucose group of each parameter. *P ≤ 0.05; **P ≤ 0.01; ***P ≤ 0.001; ****P ≤ 0.0001

Cell cycle modulation of microglial cells exposed to high glucose and treated with CA was also analyzed by flow cytometry using PI staining, which measures proliferation effects by DNA content present in cells (Fig. 10). Notably, the high glucose medium increased the population of cells in the G0/G1 phase by 60% compared to untreated control cells. Additionally, exposure to high glucose reduced the population of BV-2 cells at the S and G2/M phases (37% and 27%, respectively, Fig. 10B) compared to unexposed control cells. Moreover, the treatments with both CA concentrations (50 and 75 µM) decreased the G0/G1 phase of the cell cycle by 29% and 23%, respectively, when compared to the untreated and high glucose-challenged group. Only CA at 75 µM increased by 42% the cell population in the S phase compared to untreated cells and exposed to high glucose. Finally, BV-2 cells treated with CA (50 and 75 µM) increased by 27% and 28%, respectively, the population of cells in the G2/M phases when compared to untreated and high glucose-exposed cells (Fig. 10B).

Collectively, these findings indicate that high glucose altered cell cycle distribution and increased apoptotic cell death in BV-2 cells, whereas CA treatment partially mitigated these alterations.

## Discussion

Neurodegeneration associated with hyperglycemia-induced neuroinflammatory responses contribute to the progression of DE [6, 23, 24, 80]. Thus, to comprehend the neuroinflammatory mechanisms associated with DM, it is particularly relevant to investigate microglia as a key regulator of neuroinflammation, given the established link between the activation of these cells and cognitive impairment in DE [8, 9, 15, 24, 64]. Here, using BV-2 cells, we investigated in vitro the inflammatory responses triggered by high glucose on the set of purinergic components and showed the potential beneficial effects of CA against these glucose-elicited alterations.

In order to improve the understanding of the modulatory potential of CA against high glucose-induced toxicity, parameters related to the inflammatory and cytotoxic profile in BV-2 microglial cells were investigated. Using a glucose exposure protocol at different times and concentrations, the effect on cell viability was initially verified. Similar to our study, data previously published using the same cell line exposed to 25 mM glucose at different exposure times, exhibited an increase in cell proliferation after 24 h of exposure, and a decrease in this parameter after 48 h [63]. Additionally, in order to test the cytotoxic effect of CA and thus select appropriate concentrations to be used in this study, BV-2 cells were exposed to this phenolic acid. Thus, we observed a decrease in the viability of BV-2 cells treated with CA (100–300 µM) for a period of 24 h, and after 48 h of treatment, concentrations from 75 to 300 µM also decreased this parameter. Similarly, other research demonstrated the effect of a range of CA concentrations on the viability of human melanoma cells (SK-Mel-28), demonstrating that concentrations of CA (100, 150, and 200 µM) for a period of 24 h decreased the viability of this cell line [57].

Hyperglycemia aggravates oxidative stress events associated with microglial activation [9–11, 25], therefore, some parameters regarding this aspect were analyzed. Similar to previous literature [8, 17, 24], our results showed increased NO production in microglial cells cultured under high glucose conditions. Furthermore, evidence has shown that high glucose concentrations, which resemble hyperglycemia found in DM, increased the production of RS in BV-2 cells [17, 81]. Cognitive impairment was also associated with an increase in the levels of RS and TBARS in the hippocampus of mice in a model of neurodegeneration triggered by Aβ [49]. Furthermore, our study revealed that BV-2 cells cultured in a high glucose medium increased RS and TBARS levels. These findings support the understanding that high glucose in vitro triggers oxidative stress that can potentially damage biological membranes and genetic material. However, treatment with CA reduced the increase in the levels of both parameters in cells exposed to the high glucose medium (25 mM). Thus, the decrease in these oxidative parameters after treatment with CA may be attributed to the antioxidant capacity of this compound, which is related to its chemical structure containing hydroxyl groups associated with catechol [48, 49].

Purinergic signaling plays an important role in the CNS and dysregulation of this pathway has been linked to cellular inflammatory responses involving microglia, thus contributing to the pathogenesis of neurodegeneration [27, 28, 33, 82, 83]. The regulation of adenine nucleotides (ATP, ADP, and AMP) as well as Ado is strictly controlled by the activity of purinergic enzymes (E-NTPDase, E-5’-NT, and ADA). Consequently, alterations in the activity of these enzymes have been associated with cognitive impairment [59, 84, 85]. Our study provides evidence of alterations in purinergic enzymes of microglial cells exposed to a high glucose medium. Thus, a dysfunction in the activity of E-NTPDase and E-5’-NT may contribute to altered extracellular nucleotide metabolism and can lead to an increase in nucleotides such as ATP, which is known to be a damage-associated molecular pattern (DAMP) and a pro-inflammatory agent. Thus, these enzymatic alterations may be associated with inflammatory responses previously described under diabetic conditions [59, 85, 86].

Additionally, increased ADA activity has been associated with the diabetic state [59, 85, 86]. Since increased ADA activity may favor Ado degradation, our findings suggest that the altered ADA activity observed under high glucose conditions could potentially influence extracellular Ado availability. This is relevant because Ado participates in the regulation of several CNS functions, including cognitive activities, memory, learning, and motor functions [29, 87]. Furthermore, it is established that Ado mediates important phenomena in the brain by binding to its metabotropic receptors, particularly A1R and A2AR [47, 59, 82]. Our results demonstrated a decrease/increase in the density of A1R/A2AR receptors, respectively, in BV-2 cells exposed to high glucose. Although the mechanisms that led to changes in the density of these purinergic receptors in microglial cells are not yet well defined, it can be postulated that high glucose conditions may affect purinergic signaling and be associated with inflammatory responses that favor a more neurodegenerative environment [30, 31]. However, although the treatment with CA at 50 µM under high glucose conditions did not attenuate changes in the density of A1R (decrease) and A2AR (increase) receptors, CA at 75 µM partially reversed these alterations evoked by high glucose. These concentration-dependent differences suggest that a higher concentration of this phenolic acid may be required to modulate purinergic receptor-related changes induced by high glucose in this experimental model [51, 59].

In summary, changes in the activity of the enzymatic chain (E-NTPDase, E-5’-NT, and ADA) and in the density of the purinoceptors (A1R and A2AR) may contribute to alterations in purinergic signaling and may be associated with inflammatory responses under high glucose conditions. Nevertheless, CA treatment was able to beneficially regulate changes in purinergic enzyme activities and density of receptors. Previous studies have indicated the modulatory role of this phenolic acid in the activity of these ectoenzymes, as well as on the expression and density of purinoceptors [51, 59, 88].

Taken together, our results suggest that the modulation of purinergic signaling by CA may be associated with its broader antioxidant and anti-inflammatory effects under high glucose conditions. The restoration of E-NTPDase and E-5′-NT activities and the reduction in ADA activity may contribute to the normalization of extracellular nucleotide and adenosine metabolism. In parallel, CA restored, at least partially, the changes in A1R and A2AR receptor density and reduced the increase in P2X7R receptor density induced by high glucose, concomitantly with reductions in oxidative stress parameters and inflammatory markers. These findings suggest that the modulation of purinergic signaling observed following CA treatment may occur in association with the attenuation of oxidative and inflammatory disturbances rather than through a demonstrated direct pharmacological action on purinergic receptors. Importantly, since receptor agonism, antagonism, direct activation, or receptor-binding assays were not evaluated in the present study, a direct interaction between CA and these receptors cannot be established. Therefore, our findings support the interpretation that CA attenuates high glucose-induced alterations in purinergic signaling as part of a broader antioxidant and anti-inflammatory response.

In this scenario, in an attempt to deepen our knowledge about the inflammatory processes associated with high glucose in the CNS, we investigated some mediators related to the purinergic signaling pathway such as the P2X7R purinoceptor [30, 42]. It is established that neuroinflammatory cascades are dependent on the activation of crucial mediators, such as the NLRP3 inflammasome. The NLRP3 inflammasome is a multiprotein complex that, in response to damage, coordinates the immune processes by activating caspase-1 (CASP-1), which is required for the proteolytic cleavage of pro-IL-1β to produce mature IL-1β. Accordingly, this inflammasome plays an important role in IL-1β maturation and has been implicated in the pathophysiology and progression of DM and neurodegenerative diseases [15, 29, 89–91], since increased IL-1β levels have been associated with impaired cognitive function [81]. In this context, high concentrations of extracellular ATP, by its binding to the P2X7R, activate the NLRP3 inflammasome and trigger neuroinflammatory processes associated with neurodegeneration [5]. The fundamental role of the inflammatory cascade mediated by P2X7R/NLRP3/CASP1 in diabetes-associated cognitive impairment has been suggested previously [11]. In fact, components of this pathway have also been shown to be activated in other scenarios, such as in a model of scopolamine-induced amnesia in vivo [92], in a model of mercuric chloride (HgCl_2_) exposure in vitro using human peripheral blood mononuclear cells (PBMCs) [77], and in vitro using BV-2 cells challenged with lipopolysaccharide (LPS) [93], suggesting the critical involvement of P2X7R-mediated pro-inflammatory responses across several conditions. Notably, an increase in the expression and activation of the NLRP3 inflammasome have been reported in BV-2 cells exposed to high glucose [20, 64, 81]. Additionally, studies have demonstrated the relationship between high glucose conditions, microglial activation [9, 15], and the increased levels of inflammatory mediators such as IL-1β in microglial cell cultures [16, 17, 20, 24, 64].

High glucose levels trigger the overexpression of pro-inflammatory markers, such as IBA-1 [79]. In line with these findings, our results also demonstrated an increase in the density of IBA-1, alongside with the increase in the P2X7R/NLRP3/IL-1β axis in high glucose-challenged BV-2 cells. The aforementioned results corroborate with the notion that high glucose levels, which are characteristic of DM, contribute to the increase in microglial activation [8, 9, 17]. Thus, in view of the presented findings, it could be suggested that chronic neuroinflammation may be associated with cognitive decline found in the diabetic state. This is supported by evidence that microglial overactivation and increased inflammatory mediators such as pro-inflammatory cytokines are elevated in several neuropathological states associated with learning and memory deficits [9, 11, 16]. However, treatment with CA managed to attenuate the increase in the density of the previously mentioned parameters promoted by high glucose in our study. From these results, CA may be a promising agent to reduce inflammatory responses mediated by the P2X7R/NLRP3/IL-1β axis in high glucose environments. In accordance with this assumption, a study found that treatment with CA (50 mg/kg) was capable of reducing the expression of the IBA-1 marker and other inflammatory mediators in the brains of mice with cognitive impairment [49].

Neuroinflammation, oxidative stress, and apoptosis are core players in the pathogenesis of DE [5, 10]. In this sense, to gain further insight into the mechanisms underlying high glucose-mediated alterations in microglia cells, apoptosis and cell cycle were also investigated here. Overall, the results indicated an increase in apoptotic cell death events and a reduction in the number of proliferating cells following exposure to glucose medium (25 mM). This may be due to the increase in the percentage of BV-2 cells arrested in the G0/G1 phase, which may indicate the inability of these cells to reach the mitotic phase and proliferate. Based on these results, it is possible to infer that high glucose creates a cytotoxic environment for microglial cells, ultimately leading to cell death. In accordance with the aforementioned results, hyperglycemia has been linked to cellular senescence, indicating that high glucose levels can disrupt the cell cycle in the G1 phase [12, 94].

Similarly, the aforementioned parameters were assessed in cells exposed to high glucose (25 mM) and treated with CA (50 and 75 µM). This phenolic acid was able to reduce apoptotic cell death, the percentage of cells in the G0/G1 phase, and to increase the percentage of cells in the S and G2/M phases of the cell cycle. Contrary to our findings, higher concentrations of CA (150 and 200 µM) had an antiproliferative effect, increasing the percentage of SK-Mel-28 melanoma cells in the G0/G1 phase [57]. These divergent results may be attributed to the individual behavior of the cancerous cell line at high concentrations of CA. Therefore, based on the results, we can infer that CA has the potential to induce changes in cell cycle progression.

There are some constraints associated with the present study that should be mentioned. A limitation is the use of BV-2 cells as an immortalized microglial monoculture model. Although BV-2 cells are widely used to investigate microglial responses [95], they may differ from primary microglia in terms of gene expression, receptor profile, and functional responses, as monoculture systems do not entirely reproduce the cellular and metabolic interactions occurring in the brain. In particular, crosstalk among microglia, astrocytes, and neurons, including astrocyte-neuron metabolic coupling and the lactate shuttle, may influence microglial responses under hyperglycemic conditions [96, 97]. Therefore, our findings should be interpreted within the context of an isolated microglial model. Further studies using primary microglia, microglia-astrocyte co-culture systems, organoid models, and in vivo approaches are warranted to determine whether the observed effects of high glucose and CA are maintained in more physiologically complex systems.

In line with the above-mentioned, this study was conceived as a mechanistic follow-up to our previously published in vivo investigation [59], in which CA attenuated neuroinflammation, oxidative stress, and cognitive impairment in STZ-induced diabetic rats through the modulation of cholinergic and purinergic signaling pathways. Rather than attempting to reproduce the complexity of neuroinflammation found *in vivo*, the objective of the present study was to investigate whether high glucose-exposed BV-2 microglial cells may recapitulate some of the molecular alterations observed in vivo and to explore the cellular mechanisms that may contribute to the neuroprotective effects of CA previously demonstrated. In this sense, further explorations are necessary to understand the pathways activated by this phenolic compound.

Acknowledged that neuroinflammation is a complex tissue- and system-level process [26] that cannot be fully recapitulated in the in vitro model, additional assessments for future studies should be taken into account, such as: (1) Microglial characterization by the expression of canonical microglial markers, such as TMEM119, P2Y12R, and CX3CR1; (2) Functional assessment of P2X7R receptor activation, including YO-PRO-1 uptake; (3) Agonism/antagonism of the P2X7R 7R receptor; (4) Inflammatory markers for the immunological characterization of the model, such as Cleaved caspase-1, TNF-α, IL-6, and P2Y12R; (5) Ki67 staining for cell proliferation; (6) Adenyl cyclase (AC) activity and cyclic AMP (cAMP) levels; and (7) Nucleotides (ATP, ADP, and AMP) and nucleoside (Ado) levels.

Altogether, our findings indicate that high glucose medium (25 mM) resulted in increased activation of BV-2 microglia and alterations in purinergic signaling-related parameters, which are associated with cellular inflammatory responses that regulate microglial functions. The shift to a more activated microglial phenotype in high glucose medium can be attributed to the increase in oxidative stress parameters, including NO, RS, and TBARS, and the densities of inflammatory mediators, including IBA-1, NLRP3, and IL-β. Additionally, alterations in purinergic signaling-related parameters were demonstrated by the changes in the activity of ectoenzymes (E-NTPDase, E-5’-NT, and ADA), as well as in the density of purinoceptors (P2X7R, A1R, and A2AR). Moreover, changes in the viability, cell death and cell cycle may also be associated with these inflammatory responses. Finally, the attenuation of these alterations induced by high glucose levels in BV-2 cells treated with CA may contribute, at least partially, to the cellular effects of this phenolic acid under high glucose conditions.

**Fig. 11:**
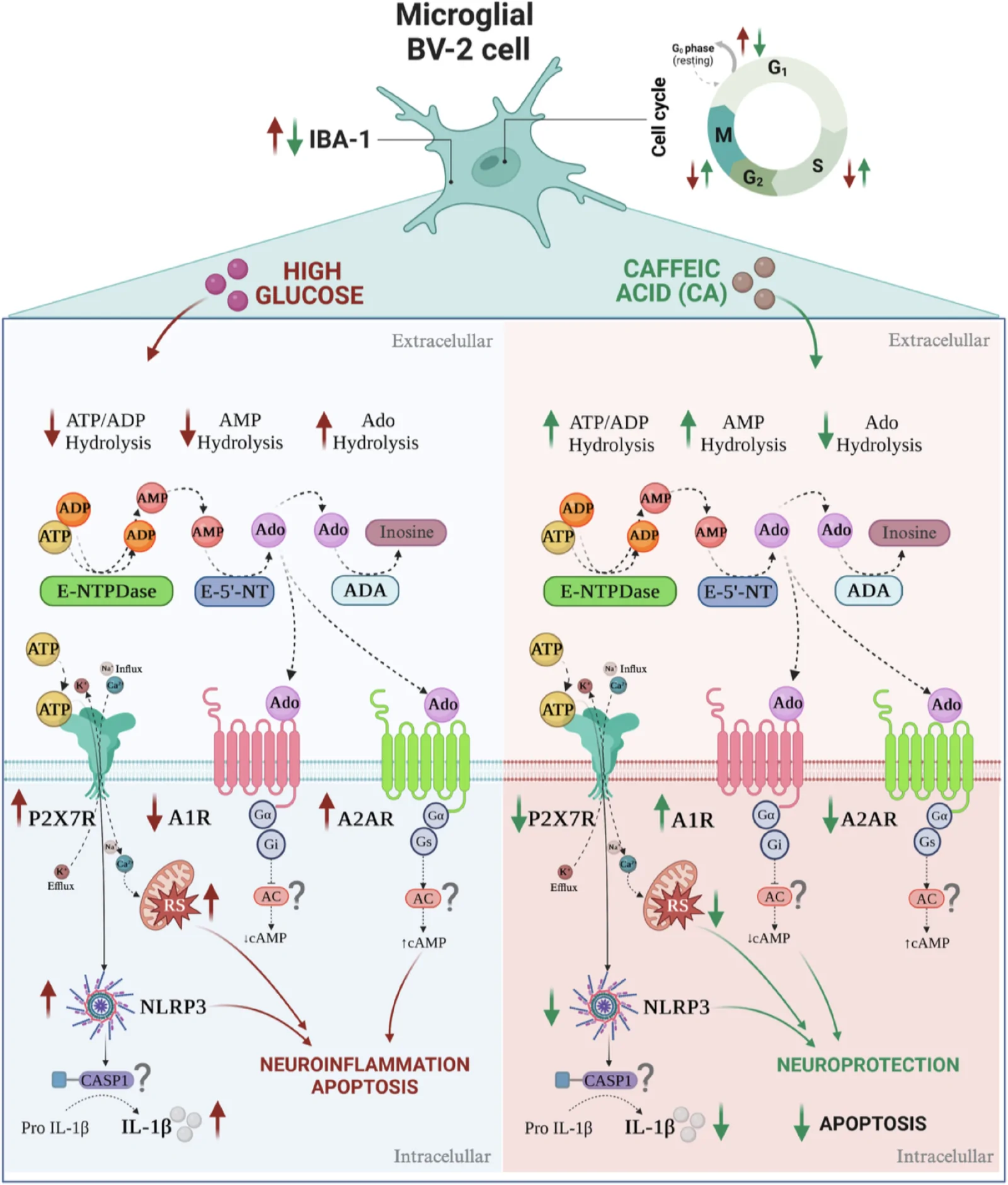
Representative figure illustrating a proposed mechanism of the treatment with CA (50 and 75 µM) on purinergic system and oxidative-inflammatory parameters in an in vitro model using BV-2 microglial cells challenged to a high glucose (25 mM) environment. High glucose has cytotoxic effects on BV-2 cells and increases the production of oxidative mediators such as nitric oxide (NO), reactive species (RS), and thiobarbituric acid reactive substances (TBARS). Alterations in the activity of purinergic system ectoenzymes, such as ecto-nucleoside triphosphate diphosphohydrolase (E-NTPDase), ecto-5’-nucleotidase (E-5’-NT), and adenosine deaminase (ADA) were also observed in high glucose-challenged cells. In addition, high glucose altered the density patterns of some purinergic receptors (P2X7R, A1R, and A2AR). Furthermore, activation of inflammatory processes with the participation of NLRP3 inflammasome and interleukin-1β (IL-1β) were demonstrated. However, treatment with CA was able to modulate these changes and cell cycle phases, as well as reduce cell death promoted by high glucose exposure. Red arrows represent the effects of high glucose exposure, whereas green arrows show the effects of the CA treatment in BV-2 microglial cells. Question marks (in gray) represent possible future mechanisms to be investigated that were not assessed here. This image was created with templates from BioRender (https://www.biorender.com)

Overall, our current findings provide a general overview of the pathways involved in the modulatory effects of CA in vitro against high glucose-challenged BV-2 cells (Fig. 11). Moreover, our results demonstrate an association between CA treatment and the possible restoration of purinergic signaling parameters under high glucose conditions, although they do not establish a direct receptor-mediated mechanism, which is open for further exploration.

## Conclusion

To sum up, our results suggest that a high glucose environment triggers microglial activation in vitro and, consequently, an inflammatory process in BV-2 cells. Overall, CA treatment mitigated changes in microglial purinergic system components, and alleviated inflammatory markers triggered by high glucose levels (Fig. 11). These findings expand the knowledge about the effects of this phenolic compound under the challenging conditions promoted by high glucose using a cellular approach which can more directly provide evidence on the protective mechanisms of bioactive molecules such as CA. Therefore, we can suggest the promising effect of CA as an adjuvant therapeutic strategy for brain health and for the treatment of inflammatory processes associated with hyperglycemia found in the diabetic state.

## Supplementary Information

Below is the link to the electronic supplementary material.

Supplementary Material 1

## Data Availability

All data are provided within the manuscript or its supplementary materials.

## References

[1] Cai J, Cai M, Xia W et al (2022) Explore the Mechanism of β-Asarone on Improving Cognitive Dysfunction in Rats with Diabetic Encephalopathy. J Alzheimer’s Dis Rep 6:195–206. 10.3233/ADR-220001 PMC9108628 35591951

[2] Ganguly R, Singh SV, Jaiswal K et al (2023) Modulatory effect of caffeic acid in alleviating diabetes and associated complications. World J Diabetes 14:62–75. 10.4239/wjd.v14.i2.62 PMC10011896 36926656

[3] Çiğ E, Yardımcı M, Çınar R, Yıldızhan K (2026) Protective role of zingerone against high glucose-Induced retinal pigment epithelial cell damage through modulation of the TRPM2 channel pathway. Mol Biol Rep 53:309. 10.1007/s11033-026-11476-9 41557015

[4] Cheon SY, Song J (2021) The Association between Hepatic Encephalopathy and Diabetic Encephalopathy: The Brain-Liver Axis. Int J Mol Sci 22:463. 10.3390/ijms22010463 PMC7796499 33466498

[5] Xu T, Liu J, Li X et al (2021) The mTOR/NF-κB Pathway Mediates Neuroinflammation and Synaptic Plasticity in Diabetic Encephalopathy. Mol Neurobiol 58:3848–3862. 10.1007/s12035-021-02390-1 33860440

[6] Cheng L, Li W, Chen Y et al (2022) Autophagy and Diabetic Encephalopathy: Mechanistic Insights and Potential Therapeutic Implications. Aging Dis 13:447. 10.14336/AD.2021.0823 PMC8947837 35371595

[7] Liu J, Deng Z, Yu Z et al (2022) The circRNA circ-Nbea participates in regulating diabetic encephalopathy. Brain Res 1774:147702. 10.1016/j.brainres.2021.147702 34695392

[8] Hininger-Favier I, Thangthaeng N, Bielinski DF et al (2021) Blueberries and insulin protect microglial cells against high glucose-induced inflammation and restore GLUT-1. J Berry Res 11:201–216. 10.3233/JBR-200628

[9] Hung H, Tsai S, Sie S, Kuo Y (2022) High glucose enhances lipopolysaccharide-induced inflammation in cultured BV2 microglial cell line. Immun Inflamm Dis 10:e610. 10.1002/iid3.610 PMC9017628 35478445

[10] Nan F, Sun G, Xie W et al (2019) Ginsenoside Rb1 mitigates oxidative stress and apoptosis induced by methylglyoxal in SH-SY5Y cells via the PI3K/Akt pathway. Mol Cell Probes 48:101469. 10.1016/j.mcp.2019.101469 31629029

[11] Jing G, Wang H, Nan F et al (2021) Naofucong ameliorates high glucose induced hippocampal neuron injury through suppressing P2X7/NLRP1/Caspase-1 pathway. Front Pharmacol 12:647116. 10.3389/fphar.2021.647116 PMC8173084 34093185

[12] Pattarachotanant N, Tencomnao T (2020) Citrus hystrix Extracts Protect Human Neuronal Cells against High Glucose-Induced Senescence. Pharmaceuticals 13:283. 10.3390/ph13100283 PMC7600454 33007805

[13] Gan W-J, Gao C-L, Zhang W-Q et al (2021) Kuwanon G protects HT22 cells from advanced glycation end product-induced damage. Exp Ther Med 21:425. 10.3892/etm.2021.9869 PMC7967837 33747164

[14] Golderman V, Goldberg Z, Gofrit SG et al (2023) PARIN5, a novel thrombin receptor antagonist modulates a streptozotocin mice model for diabetic encephalopathy. Int J Mol Sci 24:2021. 10.3390/ijms24032021 PMC9917200 36768341

[15] Zhang X, Huang S, Zhuang Z et al (2023) Lipin2 ameliorates diabetic encephalopathy via suppressing JNK/ERK-mediated NLRP3 inflammasome overactivation. Int Immunopharmacol 118:109930. 10.1016/j.intimp.2023.109930 37001383

[16] Jing G, Zhang M, Ji C et al (2016) Effect of Chinese herbal compound Naofucong (脑复聪) on the inflammatory process induced by high glucose in BV-2 cells. Chin J Integr Med 22:832–839. 10.1007/s11655-016-2256-0 27225293

[17] Kongtawelert P, Kaewmool C, Phitak T et al (2022) Sesamin protects against neurotoxicity via inhibition of microglial activation under high glucose circumstances through modulating p38 and JNK signaling pathways. Sci Rep 12:11296. 10.1038/s41598-022-15411-3 PMC9253356 35788665

[18] Dadwal S, Heneka MT (2024) Microglia heterogeneity in health and disease. FEBS Open Bio 14:217–229. 10.1002/2211-5463.13735 PMC10839410 37945346

[19] Flury A, St-Pierre M-K, Ayata P, Tremblay M-È (2026) Evolving insights into the identity and function of dark microglia. Trends Neurosci 49:20–34. 10.1016/j.tins.2025.11.002 PMC12695070 41354574

[20] Li Y, Long W, Gao M et al (2021) TREM2 Regulates High Glucose-Induced Microglial Inflammation via the NLRP3 Signaling Pathway. Brain Sci 11:896. 10.3390/brainsci11070896 PMC8306970 34356130

[21] Han R, Liu Z, Sun N et al (2019) BDNF Alleviates Neuroinflammation in the Hippocampus of Type 1 Diabetic Mice via Blocking the Aberrant HMGB1/RAGE/NF-κB Pathway. Aging Dis 10:611. 10.14336/AD.2018.0707 PMC6538223 31165005

[22] Waltrick APF, da Silva ACF, de Mattos BA et al (2023) Long-term treatment with roflumilast improves learning of fear extinction memory and anxiety-like response in a type-1 diabetes mellitus animal model. Behav Brain Res 439:114217. 10.1016/j.bbr.2022.114217 36400237

[23] Maher P (2021) Investigations into the Role of Metabolism in the Inflammatory Response of BV2 Microglial Cells. Antioxidants 10:109. 10.3390/antiox10010109 PMC7828726 33466581

[24] Iannucci J, Rao HV, Grammas P (2022) High Glucose and Hypoxia-Mediated Damage to Human Brain Microvessel Endothelial Cells Induces an Altered, Pro-Inflammatory Phenotype in BV-2 Microglia In Vitro. Cell Mol Neurobiol 42:985–996. 10.1007/s10571-020-00987-z PMC8942976 33136275

[25] DiSabato DJ, Quan N, Godbout JP (2016) Neuroinflammation: the devil is in the details. J Neurochem 139:136–153. 10.1111/jnc.13607 PMC5025335 26990767

[26] Müller L, Di Benedetto S, Müller V (2025) From homeostasis to neuroinflammation: insights into cellular and molecular interactions and network dynamics. Cells 14:54. 10.3390/cells14010054 PMC11720143 39791755

[27] Aminin D, Illes P (2021) Purinergic signaling in neuroinflammation. Int J Mol Sci 22:12895. 10.3390/ijms222312895 PMC8657746 34884700

[28] Assmann CE, Apolloni S, Ignácio ZM, Bagatini MD (2022) Editorial: purinergic signaling and neuroinflammation. Front Pharmacol 13:1113063. 10.3389/fphar.2022.1113063 PMC9798437 36588721

[29] Beamer E, Gölöncsér F, Horváth G et al (2016) Purinergic mechanisms in neuroinflammation: an update from molecules to behavior. Neuropharmacology 104:94–104. 10.1016/j.neuropharm.2015.09.019 26384652

[30] Carracedo S, Launay A, Dechelle-Marquet P-A et al (2024) Purinergic-associated immune responses in neurodegenerative diseases. Prog Neurobiol 243:102693. 10.1016/j.pneurobio.2024.102693 39579963

[31] Pietrowski MJ, Gabr AA, Kozlov S et al (2021) Glial purinergic signaling in neurodegeneration. Front Neurol 12:654850. 10.3389/fneur.2021.654850 PMC8160300 34054698

[32] Bours MJL, Swennen ELR, Di Virgilio F et al (2006) Adenosine 5′-triphosphate and adenosine as endogenous signaling molecules in immunity and inflammation. Pharmacol Ther 112:358–404. 10.1016/j.pharmthera.2005.04.013 16784779

[33] Huang Z, Xie N, Illes P et al (2021) From purines to purinergic signalling: molecular functions and human diseases. Signal Transduct Target Ther 6:162. 10.1038/s41392-021-00553-z PMC8079716 33907179

[34] von Kügelgen I (2024) Pharmacological characterization of P2Y receptor subtypes – an update. Purinergic Signal 20:99–108. 10.1007/s11302-023-09963-w PMC10997570 37697211

[35] Robson SC, Sévigny J, Zimmermann H (2006) The E-NTPDase family of ectonucleotidases: Structure function relationships and pathophysiological significance. Purinergic Signal 2:409–430. 10.1007/s11302-006-9003-5 PMC2254478 18404480

[36] Yegutkin GG (2008) Nucleotide- and nucleoside-converting ectoenzymes: Important modulators of purinergic signalling cascade. Biochim Biophys Acta - Mol Cell Res 1783:673–694. 10.1016/j.bbamcr.2008.01.024 18302942

[37] Junger WG (2011) Immune cell regulation by autocrine purinergic signalling. Nat Rev Immunol 11:201–212. 10.1038/nri2938 PMC4209705 21331080

[38] Reichert KP, Castro MFV, Assmann CE et al (2021) Diabetes and hypertension: pivotal involvement of purinergic signaling. Biomed Pharmacother 137:111273. 10.1016/j.biopha.2021.111273 PMC7846467 33524787

[39] Tozzi M, Larsen AT, Lange SC et al (2018) The P2X7 receptor and pannexin-1 are involved in glucose-induced autocrine regulation in β-cells. Sci Rep 8:8926. 10.1038/s41598-018-27281-9 PMC5997690 29895988

[40] Morais-Lima TM, Vicentini JC, Alberto AVP et al (2020) The role of purinergic signaling in the pathophysiology of perinatal hypoxic-ischemic encephalopathy. Receptors P1 and P2 as targets for drug therapy in humans. IntechOpen, London. 10.5772/intechopen.86425

[41] Tassetto M, Scialdone A, Solini A, Di Virgilio F (2021) The P2X7 receptor: a promising pharmacological target in diabetic retinopathy. Int J Mol Sci 22:7110. 10.3390/ijms22137110 PMC8268192 34281162

[42] Di Virgilio F (2007) Liaisons dangereuses: P2X7 and the inflammasome. Trends Pharmacol Sci 28:465–472. 10.1016/j.tips.2007.07.002 17692395

[43] Cunha RA (2016) How does adenosine control neuronal dysfunction and neurodegeneration? J Neurochem 139:1019–1055. 10.1111/jnc.13724 27365148

[44] Kumar V, Sharma A (2009) Adenosine: An endogenous modulator of innate immune system with therapeutic potential. Eur J Pharmacol 616:7–15. 10.1016/j.ejphar.2009.05.005 19464286

[45] Fredholm BB, IJzerman AP, Jacobson KA et al (2011) International Union of Basic and Clinical Pharmacology. LXXXI. Nomenclature and Classification of Adenosine Receptors—An Update. Pharmacol Rev 63:1–34. 10.1124/pr.110.003285 PMC3061413 21303899

[46] Jang M-H, Song J (2024) Adenosine and adenosine receptors in metabolic imbalance-related neurological issues. Biomed Pharmacother 177:116996. 10.1016/j.biopha.2024.116996 PMC12021433 38897158

[47] Cunha RA (2005) Neuroprotection by adenosine in the brain: From A1 receptor activation to A2A receptor blockade. Purinergic Signal 1:111–134. 10.1007/s11302-005-0649-1 PMC2096528 18404497

[48] Espíndola KMM, Ferreira RG, Narvaez LEM et al (2019) Chemical and Pharmacological Aspects of Caffeic Acid and Its Activity in Hepatocarcinoma. Front Oncol 9:541. 10.3389/fonc.2019.00541 PMC6598430 31293975

[49] Khan A, Park JS, Kang MH et al (2023) Caffeic acid, a polyphenolic micronutrient rescues mice brains against Aβ-induced neurodegeneration and memory impairment. Antioxidants (Basel) 12:1284. 10.3390/antiox12061284 PMC10295243 37372012

[50] Oršolić N, Sirovina D, Odeh D et al (2021) Efficacy of caffeic acid on diabetes and its complications in the mouse. Molecules 26:3262. 10.3390/molecules26113262 PMC8199327 34071554

[51] Castro MFV, Stefanello N, Assmann CE et al (2021) Modulatory effects of caffeic acid on purinergic and cholinergic systems and oxi-inflammatory parameters of streptozotocin-induced diabetic rats. Life Sci 277:119421. 10.1016/j.lfs.2021.119421 33785337

[52] Hsu F-L, Chen Y-C, Cheng J-T (2000) Caffeic Acid as Active Principle from the Fruit of Xanthiumstrumarium to Lower Plasma Glucose in Diabetic Rats. Planta Med 66:228–230. 10.1055/s-2000-8561 10821047

[53] Jung UJ, Lee M-K, Park YB et al (2006) Antihyperglycemic and antioxidant properties of caffeic acid in db/db mice. J Pharmacol Exp Ther 318:476–483. 10.1124/jpet.106.105163 16644902

[54] Akhlaghipour I, Nasimi Shad A, Askari VR et al (2023) How caffeic acid and its derivatives combat diabetes and its complications: a systematic review. J Funct Foods 110:105862. 10.1016/j.jff.2023.105862

[55] da Bonadiman BSR, Weis GCC, da Rosa JR et al (2021) Effects of caffeic acid on oxidative balance and cancer. Cancer. Elsevier, Amsterdam, pp 291–300

[56] Pavlíková N (2022) Caffeic acid and diseases—mechanisms of action. Int J Mol Sci 24:588. 10.3390/ijms24010588 PMC9820408 36614030

[57] Pelinson LP, Assmann CE, Palma TV et al (2019) Antiproliferative and apoptotic effects of caffeic acid on SK-Mel-28 human melanoma cancer cells. Mol Biol Rep 46:2085–2092. 10.1007/s11033-019-04658-1 30719606

[58] Wang Y, Wang Y, Li J et al (2016) Effects of caffeic acid on learning deficits in a model of Alzheimer’s disease. Int J Mol Med 38:869–875. 10.3892/ijmm.2016.2683 27430591

[59] Castro MFV, Assmann CE, Stefanello N et al (2023) Caffeic acid attenuates neuroinflammation and cognitive impairment in streptozotocin-induced diabetic rats: pivotal role of the cholinergic and purinergic signaling pathways. J Nutr Biochem 115:109280. 10.1016/j.jnutbio.2023.109280 36796549

[60] Deshmukh R, Kaundal M, Bansal V, Samardeep (2016) Caffeic acid attenuates oxidative stress, learning and memory deficit in intra-cerebroventricular streptozotocin induced experimental dementia in rats. Biomed Pharmacother 81:56–62. 10.1016/j.biopha.2016.03.017 27261577

[61] Sirichoat A, Dornlakorn O, Saenno R et al (2024) Caffeic acid protects against l-methionine induced reduction in neurogenesis and cognitive impairment in a rat model. Heliyon 10:e26919. 10.1016/j.heliyon.2024.e26919 PMC10918208 38455532

[62] Assmann CE, Mostardeiro VB, Weis GCC et al (2021) Aluminum-induced alterations in purinergic system parameters of BV-2 brain microglial cells. J Immunol Res 2021:1–10. 10.1155/2021/2695490 PMC7837790 33532505

[63] Hsieh C-F, Liu C-K, Lee C-T et al (2019) Acute glucose fluctuation impacts microglial activity, leading to inflammatory activation or self-degradation. Sci Rep 9:840. 10.1038/s41598-018-37215-0 PMC6351546 30696869

[64] Zhang Y, Wang L, Guo H et al (2023) Levetiracetam attenuates diabetes-associated cognitive impairment and microglia polarization by suppressing neuroinflammation. Front Pharmacol 14:1145819. 10.3389/fphar.2023.1145819 PMC10192710 37214458

[65] Mei X, Zhang T, Ouyang H et al (2019) Scutellarin alleviates blood-retina-barrier oxidative stress injury initiated by activated microglia cells during the development of diabetic retinopathy. Biochem Pharmacol 159:82–95. 10.1016/j.bcp.2018.11.011 30447218

[66] Videtta G, Sasia C, Galeotti N (2025) High Rosmarinic Acid Content Melissa officinalis L. Phytocomplex Modulates Microglia Neuroinflammation Induced by High Glucose. Antioxidants 14:161. 10.3390/antiox14020161 PMC11851730 40002348

[67] Zhang T, Mei X, Ouyang H et al (2019) Natural flavonoid galangin alleviates microglia-trigged blood–retinal barrier dysfunction during the development of diabetic retinopathy. J Nutr Biochem 65:1–14. 10.1016/j.jnutbio.2018.11.006 30597356

[68] Mosmann T (1983) Rapid colorimetric assay for cellular growth and survival: application to proliferation and cytotoxicity assays. J Immunol Methods 65:55–63. 10.1016/0022-1759(83)90303-4 6606682

[69] Grisham MB, Johnson GG, Lancaster JR (1996) Quantitation of nitrate and nitrite in extracellular fluids. Methods in enzymology. Elsevier, Amsterdam, pp 237–246. 10.1016/s0076-6879(96)68026-4 8782590

[70] Ohkawa H, Ohishi N, Yagi K (1979) Assay for lipid peroxides in animal tissues by thiobarbituric acid reaction. Anal Biochem 95:351–358. 10.1016/0003-2697(79)90738-3 36810

[71] Halliwell B, Whiteman M (2004) Measuring reactive species and oxidative damage in vivo and in cell culture: How should you do it and what do the results mean? Br J Pharmacol 142:231–255. 10.1038/sj.bjp.0705776 PMC1574951 15155533

[72] Schetinger MRC, Porto NM, Moretto MB et al (2000) New Benzodiazepines Alter Acetylcholinesterase and ATPDase Activities. Neurochem Res 25:949–955. 10.1023/A:1007500424392 10959491

[73] Heymann D, Reddington M, Kreutzberg GW (1984) Subcellular localization of 5′-nucleotidase in rat brain. J Neurochem 43:971–978. 10.1111/j.1471-4159.1984.tb12832.x 6088700

[74] Giusti G, Galanti B (1984) Adenosine deaminase: colorimetric method. In: Bergmeyer H-U (ed) Methods of Enzymatic Analysis, 3rd ed. Verlag Chemie, pp 315–323

[75] Mostardeiro VB, Assmann CE, Schirmann AA et al (2025) Quercetin and Tranylcypromine Improve Memory, Behavioral Performance, and Cholinergic Function in Male Rats Subjected to Chronic Restraint Stress. Brain Sci 15:709. 10.3390/brainsci15070709 PMC12293918 40722302

[76] Assmann CE, Cadoná FC, Bonadiman B et al (2018) Tea tree oil presents in vitro antitumor activity on breast cancer cells without cytotoxic effects on fibroblasts and on peripheral blood mononuclear cells. Biomed Pharmacother 103:1253–1261. 10.1016/j.biopha.2018.04.096 29864906

[77] Schott M, Assmann CE, Rambo BVC et al (2025) Cytotoxic and immunotoxic profile of HgCl2 involves alterations in purinergic signaling through the P2X7/NLRP3/CASP-1/IL-1β pathway: An in vitro study using human blood immune cells. Toxicol Vitr 109:106131. 10.1016/j.tiv.2025.106131 40840770

[78] Bradford MM (1976) A rapid and sensitive method for the quantitation of microgram quantities of protein utilizing the principle of protein-dye binding. Anal Biochem 72:248–254. 10.1016/0003-2697(76)90527-3 942051

[79] Vargas-Soria M, García-Alloza M, Corraliza-Gómez M (2023) Effects of diabetes on microglial physiology: a systematic review of in vitro, preclinical and clinical studies. J Neuroinflammation 20:57. 10.1186/s12974-023-02740-x PMC9983227 36869375

[80] Li X, Yu P, Yu Y et al (2021) Hydrogen sulfide ameliorates high glucose-induced pro-inflammation factors in HT-22 cells: involvement of SIRT1-mTOR/NF-κB signaling pathway. Int Immunopharmacol 95:107545. 10.1016/j.intimp.2021.107545 33765609

[81] Tian R, Liu X, Xiao Y et al (2024) Huang-Lian-Jie-Du decoction drug-containing serum inhibits IL-1β secretion from D-glucose and PA induced BV2 cells via autophagy/NLRP3 signaling. J Ethnopharmacol 323:117686. 10.1016/j.jep.2023.117686 38160864

[82] Bagatini MD, dos Santos AA, Cardoso AM et al (2018) The impact of purinergic system enzymes on noncommunicable, neurological, and degenerative diseases. J Immunol Res 2018:1–21. 10.1155/2018/4892473 PMC6109496 30159340

[83] Zarrinmayeh H, Territo PR (2020) Purinergic receptors of the central nervous system: biology, PET ligands, and their applications. Mol Imaging 19:1536012120927609. 10.1177/1536012120927609 PMC7297484 32539522

[84] Gutierres JM, Carvalho FB, Schetinger MRC et al (2012) Protective effects of anthocyanins on the ectonucleotidase activity in the impairment of memory induced by scopolamine in adult rats. Life Sci 91:1221–1228. 10.1016/j.lfs.2012.09.013 23044227

[85] Maciel RM, Carvalho FB, Olabiyi AA et al (2016) Neuroprotective effects of quercetin on memory and anxiogenic-like behavior in diabetic rats: Role of ectonucleotidases and acetylcholinesterase activities. Biomed Pharmacother 84:559–568. 10.1016/j.biopha.2016.09.069 27694000

[86] Fotino C, Dal Ben D, Adinolfi E (2018) Emerging Roles of Purinergic Signaling in Diabetes. Med Chem (Los Angeles) 14:428–438. 10.2174/1573406414666180226165204 29485002

[87] Huo M, Wang Z, Fu W et al (2021) Spatially Resolved Metabolomics Based on Air-Flow-Assisted Desorption Electrospray Ionization–Mass Spectrometry Imaging Reveals Region-Specific Metabolic Alterations in Diabetic Encephalopathy. J Proteome Res 20:3567–3579. 10.1021/acs.jproteome.1c00179 34137614

[88] Anwar J, Spanevello RM, Pimentel VC et al (2013) Caffeic acid treatment alters the extracellular adenine nucleotide hydrolysis in platelets and lymphocytes of adult rats. Food Chem Toxicol 56:459–466. 10.1016/j.fct.2013.02.030 23454297

[89] Nițulescu IM, Ciulei G, Cozma A et al (2023) From Innate Immunity to Metabolic Disorder: A Review of the NLRP3 Inflammasome in Diabetes Mellitus. J Clin Med 12:6022. 10.3390/jcm12186022 PMC10531881 37762961

[90] Ward R, Li W, Abdul Y et al (2019) NLRP3 inflammasome inhibition with MCC950 improves diabetes-mediated cognitive impairment and vasoneuronal remodeling after ischemia. Pharmacol Res 142:237–250. 10.1016/j.phrs.2019.01.035 PMC6486792 30818045

[91] Zhai Y, Meng X, Ye T et al (2018) Inhibiting the NLRP3 Inflammasome Activation with MCC950 Ameliorates Diabetic Encephalopathy in db/db Mice. Molecules 23:522. 10.3390/molecules23030522 PMC6017493 29495433

[92] da Costa P, Schetinger MRC, Baldissarelli J et al (2025) Blackcurrant (*Ribes nigrum* L.) and its association with donepezil restore cognitive impairment, suppress oxidative stress and pro-inflammatory responses, and improve purinergic signaling in a scopolamine-induced amnesia model in mice. Neurochem Res 50:79. 10.1007/s11064-024-04327-1 39800790

[93] Rambo BVC, Castro MFV, Schott M et al (2026) Capsaicin suppresses LPS-induced inflammatory responses via NLRP3/CASP-1/IL-1β axis and purinergic pathways in BV-2 microglial cells. Purinergic Signal 22:3. 10.1007/s11302-025-10123-5 PMC12796030 41524965

[94] Mao Z, Ke Z, Gorbunova V, Seluanov A (2012) Replicatively senescent cells are arrested in G1 and G2 phases. Aging 4:431–435. 10.18632/aging.100467 PMC3409679 22745179

[95] Henn A (2009) The suitability of BV2 cells as alternative model system for primary microglia cultures or for animal experiments examining brain inflammation. Altex 26:83–94. 10.14573/altex.2009.2.83 19565166

[96] Henn RE, Noureldein MH, Elzinga SE et al (2022) Glial-neuron crosstalk in health and disease: a focus on metabolism, obesity, and cognitive impairment. Neurobiol Dis 170:105766. 10.1016/j.nbd.2022.105766 PMC10071699 35584728

[97] Wang S, Wang S, Chen H, Xu J (2026) Microglia–Neuron Crosstalk: An Intimate Molecular Conversation in Neurodegeneration. Int J Mol Sci 27:2011. 10.3390/ijms27042011 PMC12940662 41752147

