# Supplementary material for: Caffeic Acid Regulates High Glucose-Induced Inflammatory Responses in BV-2 Microglial Cells: Implications for Purinergic Signaling
Source: Neurochem Res. 2026 Oct 31;51(6):315. doi: 10.1007/s11064-026-04893-6 (PMC13722727; doi:10.1007/s11064-026-04893-6)
Supplement: Supplementary file 1 [file 11064_2026_4893_MOESM1_ESM.pdf]

Supplementary material

Caffeic acid regulates high glucose-induced inflammatory responses in BV-2 microglial cells: implications for purinergic signaling

Milagros Fanny Vera Castro, Charles Elias Assmann, Bianca Vedoin Copês Rambo, Mairin Schott, Marcylene Vieira da Silveira, Vitor Bastianello Mostardeiro, Adriel Antonio Schirmann, Pâmela de Almeida Milioni, Michele Goulart dos Santos, Vera Maria Melchiors Morsch, Roselia Maria Spanevello, Maria Rosa Chitolina Schetinger

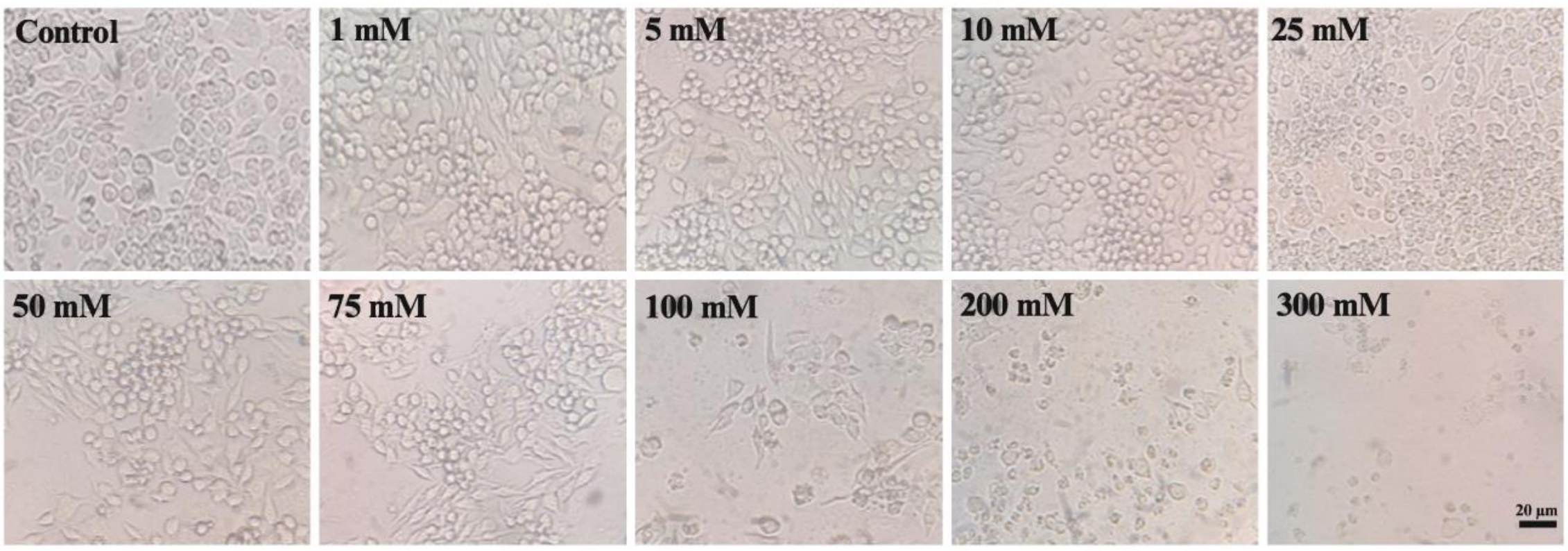

Figure S1 – **Representative morphology of BV-2 microglial cells after exposure *in vitro* to different concentrations of Glucose (1 – 300 mM) for 48 h.** The control group represents cells cultured only in medium and did not receive glucose treatment. Magnification = 40x, Scale Bar = 20 µm.

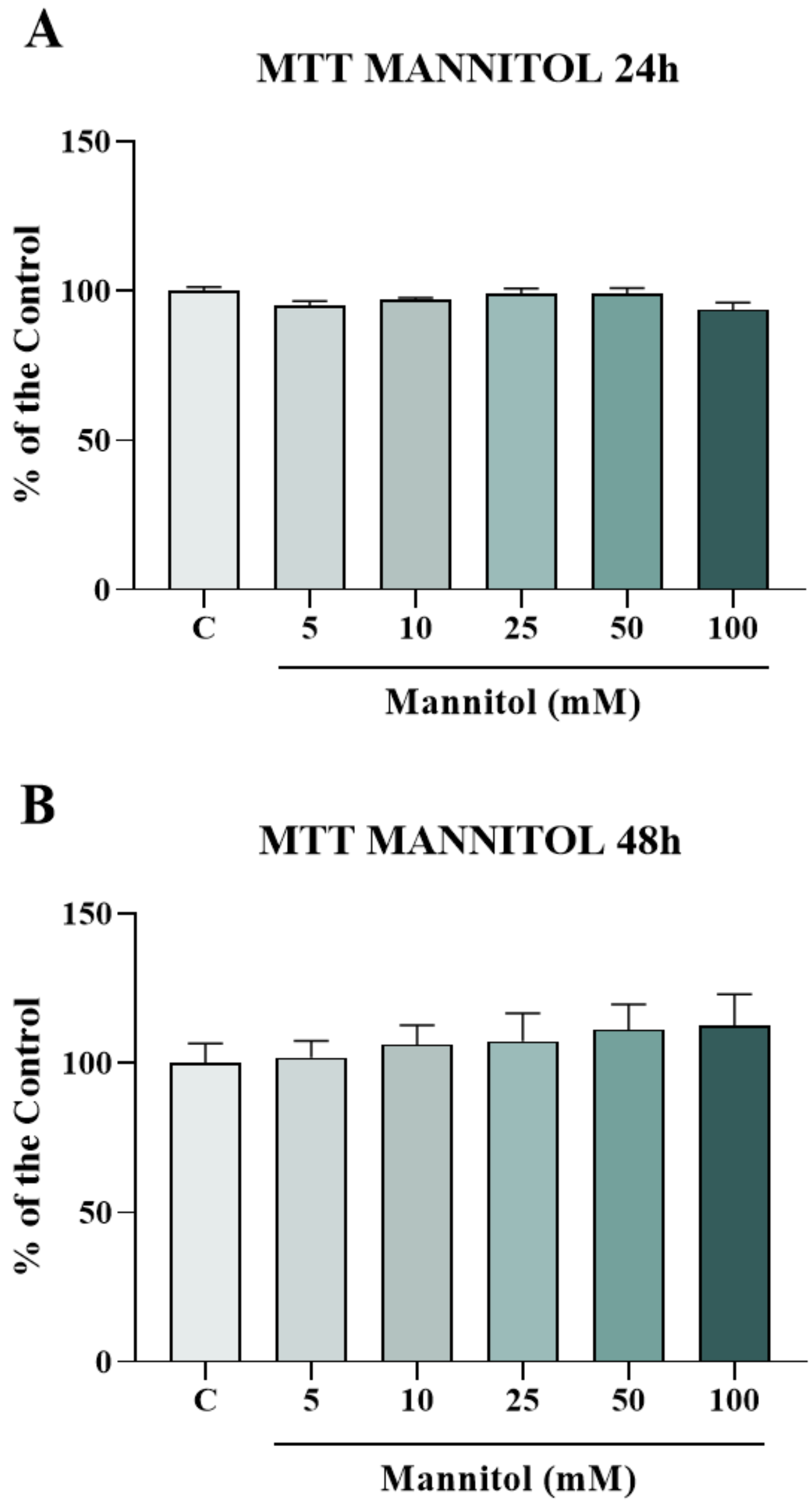

Figure S2 – Cell viability and proliferation of BV-2 microglial cells exposed *in vitro* to the osmotic control (mannitol) at concentrations of 5 – 100 mM for 24 h (A) and 48 h (B). Cell viability and proliferation were assessed by the MTT assay. C = Control group. Results are expressed as a percentage of the control group. Bars represent means ± SEM. *n* = 3. One-way ANOVA was followed by Bonferroni's post-test.

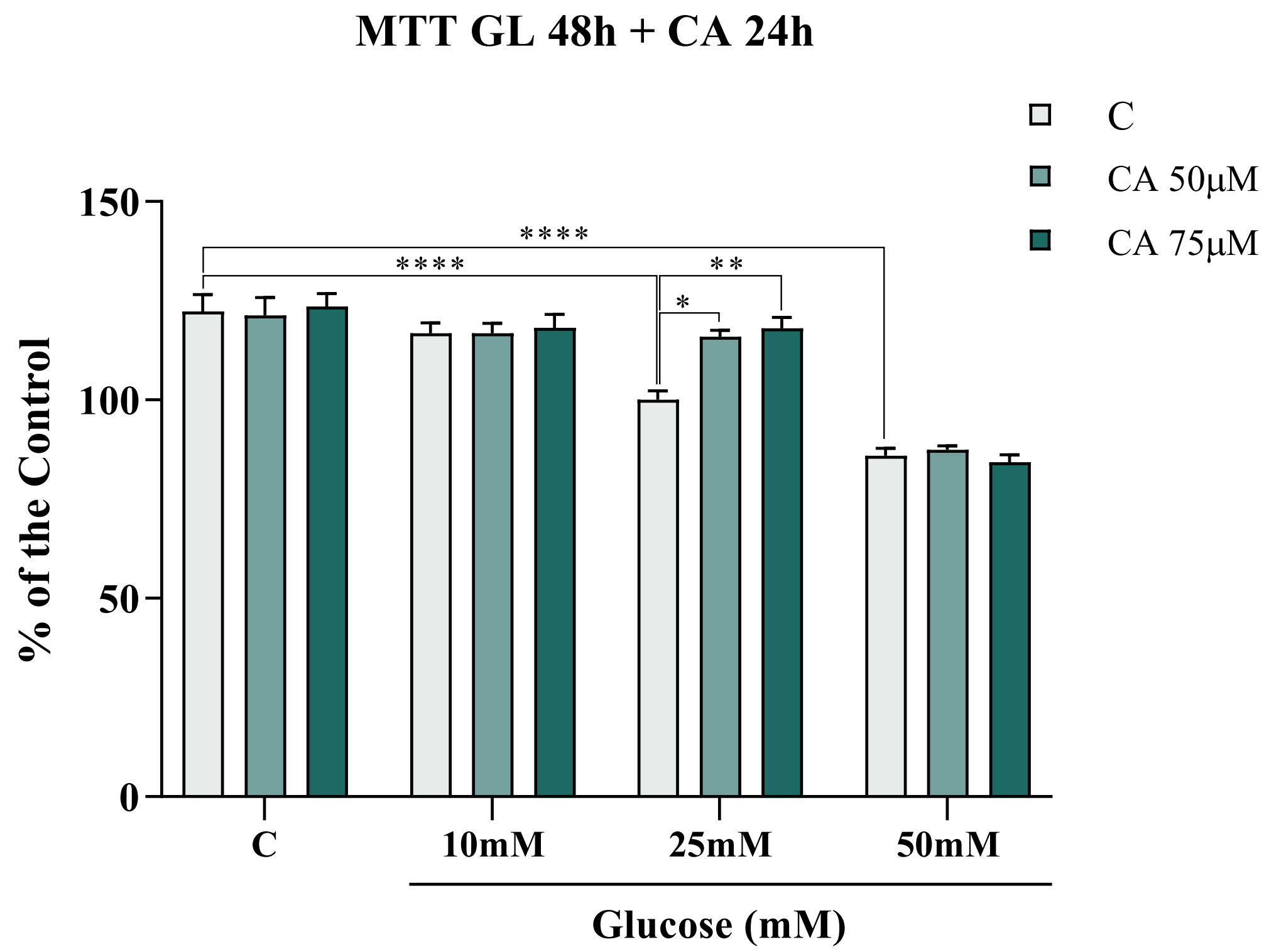

Figure S3 – Cell viability and proliferation of BV-2 microglial cells exposed *in vitro* to glucose (10, 25 and 50 mM) during 48 h and treated with CA (50 and 75 µM) for 24 h. Cell viability and proliferation were assessed by the MTT assay. C = Control group; CA = Caffeic Acid; GL = Glucose group. Results are expressed as a percentage of the control group. Bars represent means ± SEM. Two-way ANOVA was followed by Bonferroni's post-test. Significant values are considered at $P \leq 0.05$, $n = 8$. * indicates $P \leq 0.05$; ** $P \leq 0.01$; *** $P \leq 0.001$; **** $P \leq 0.0001$.

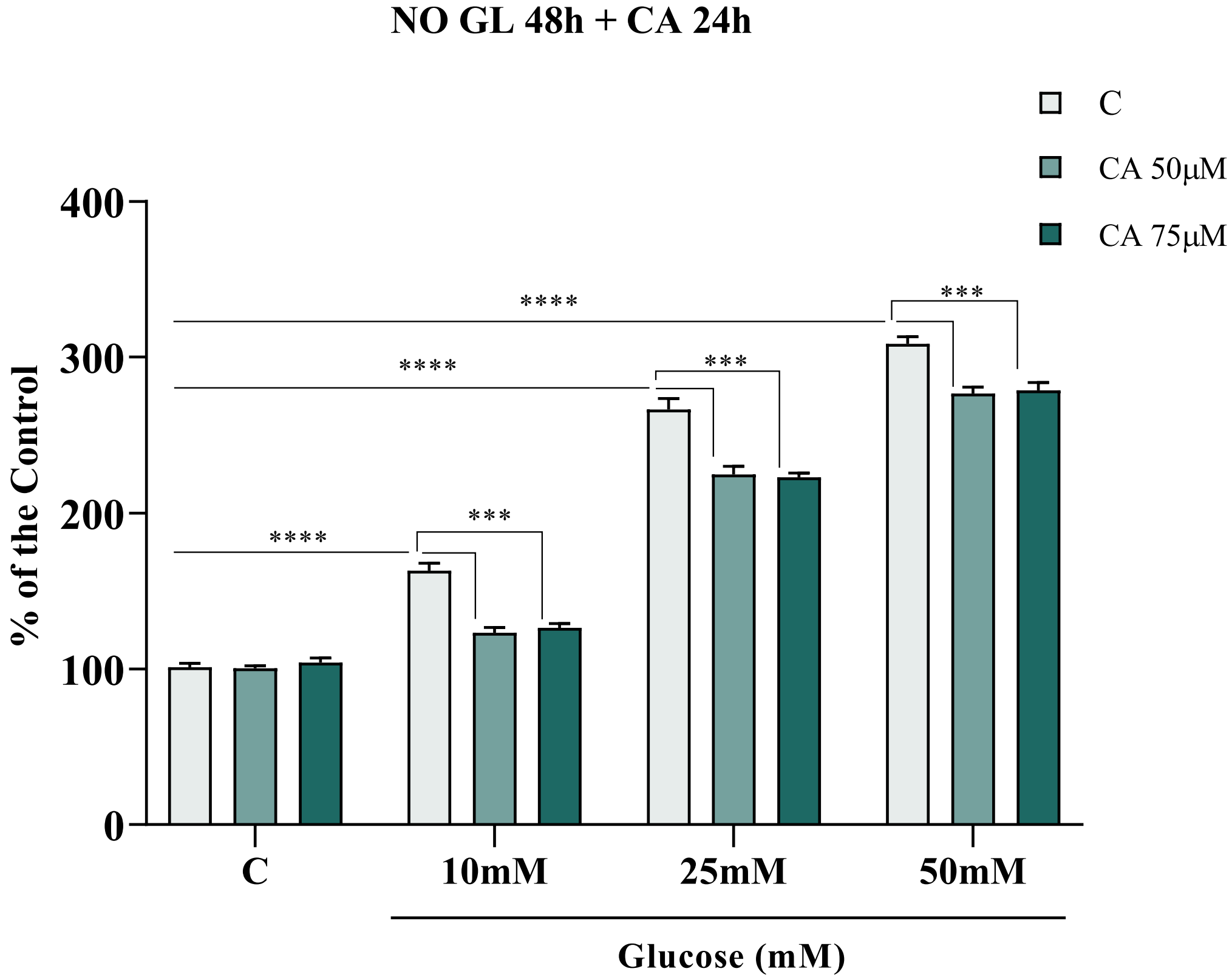

__Figure S4__ **– Nitric oxide (NO) levels of BV-2 microglial cells exposed *in vitro* to glucose (10, 25 and 50 mM) during 48 h and treated with CA (50 and 75 µM) for 24 h.** NO was assessed indirectly by nitrite levels using the Griess reagent. C = Control group; CA = Caffeic Acid; GL = Glucose group. Results are expressed as a percentage of the control group. Bars represent means ± SEM. Two-way ANOVA was followed by Bonferroni's post-test. Significant values are considered at $P \leq 0.05$, $n = 8$. * indicates $P \leq 0.05$; ** $P \leq 0.01$; *** $P \leq 0.001$; **** $P \leq 0.0001$.
